# A review on microplastics: sources, environmental fate, degradation pathways, and analytical identification methods

**DOI:** 10.1039/d5ra09062h

**Published:** 2026-03-12

**Authors:** Kavitha Kamalasekaran, Ashok K. Sundramoorthy

**Affiliations:** a Department of Chemistry, Velammal Engineering College Chennai 600066 Tamil Nadu India; b Centre for Nano-Biosensors, Department of Prosthodontics & Materials Science, Saveetha Dental College and Hospitals, Saveetha Institute of Medical and Technical Sciences Chennai Tamil Nadu 600077 India ashok.sundramoorthy@gmail.com

## Abstract

Microplastics pose a serious threat to ecosystems, making their detection and characterization a critical area of scientific research. This review provides a comprehensive overview of the current techniques employed for investigating the contamination, sources, environmental distribution, toxicological impacts, and biodegradation processes of microplastics within complex environmental matrices. A range of analytical methods is discussed, including visual inspection, microscopy techniques, and advanced spectroscopic methods such as Fourier transform infrared (FTIR) spectroscopy, Raman spectroscopy, surface-enhanced Raman spectroscopy (SERS), and quadrupole mass spectrometry (MS) coupled with pyrolysis gas chromatography. Additional approaches such as staining methods, thermogravimetric analysis (TGA), differential scanning calorimetry (DSC), and near-infrared (NIR) spectroscopy are also evaluated. Special emphasis is placed on the emerging potential of electrochemical sensing technologies as low-cost, efficient instruments for identifying and classifying microplastics. Despite the growing interest in the electrochemical remediation of microplastics, there is a notable gap in research focusing on electrochemical sensors for monitoring microplastics. The novel approach of this work is the systematic comparison and critical evaluation of various electrochemical sensing approaches for microplastic detection. This analysis is based on the most recent literature and examines their relative advantages, limitations, and suitability in comparison to conventional detection methods. Additionally, this review presents a thorough examination of strategies for the fabrication of electrochemical sensors, encompassing recognition elements, advanced immobilization techniques, and limit of detection protocols that are specifically designed for microplastic detection applications.

## Abstract 2

The review examines microplastic contamination, sources, distribution, toxicity, and biodegradation, details analytical methods, and highlights advances in electrochemical sensor design for detecting microplastics in complex environments.

## Graphical abstract

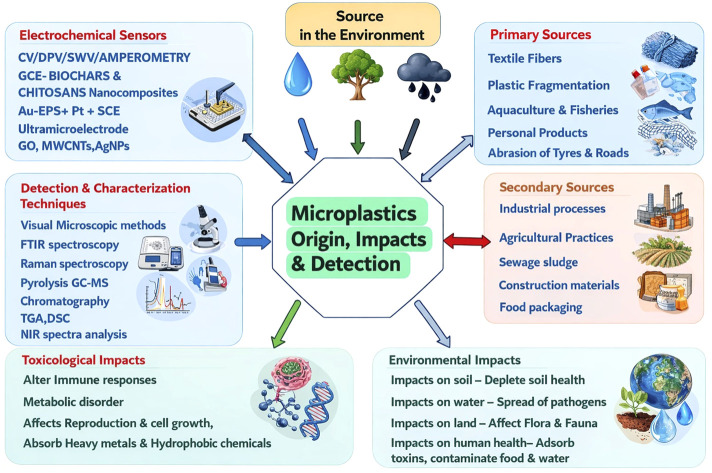

## 1 Introduction

Each year, countless tonnes of rubbish are produced as a result of the overproduction of plastics and their careless use, which frequently ends up in the air, land, aquatic environments.^1,2^ This trash is mostly polymers that linger in soil or water bodies, which frequently break down due to weathering.^3^ Environmental plastic residues interact and change into microplastics (MPs) and nanoplastics (NPs) by mechanical abrasion, heat and ultraviolet (UV) disintegration, oxidation by light, bio decay, and mechanical tension.^4–6^ Recently, microplastics have been discovered as pollutants in the entire ecosystem, including both terrestrial and aquatic environments.

Concerns are also rising over unsustainable habits, including poor waste management, inadequate recycling, and marine littering.^7^ The frequent usage and possible health risks of MPs in this environment, especially to humans, have garnered considerable interest.^8,9^ MPs are extensively dispersed throughout the ecosystems, as demonstrated by their significant quantities found in soil, air, and surface water, as well as in remote polar regions and the depths of the oceans.^10–12^ Furthermore, microplastics have been found in everyday items such as beer,^13^ sea salt,^14^ sugar,^15^ honey,^16^ tap water,^17^ bottled water,^18^ and plastic tea bags.^19^ Drawing on these sources, this article synthesizes the current pollution conditions and transport and bio-degradation mechanisms (bio-splitting, biofouling, aggregation, and long-range transport) and identifies persistent research gaps and future directions for standardized methods, quantification at the sub-micron scale, toxicity pathways, and cross-compartment mass-balance assessments.

Tiny microplastics are more easily absorbed by species and are more widely dispersed in the environment than big plastics. Microplastics are frequently transmitted through the food chain in aquatic organisms such as shellfish and seafood.^20^ In recent times, MPs have been recognised as a significant ocean pollution problem. According to Lebreton *et al.*,^21^ the majority of MPs originate on the continent and mostly reach the marine environment through rivers. Potential sources of marine microplastics include industrial and urban waste, the erosion of beach sediments, and debris from maritime activities including tourism, commercial fishing, and offshore oil and gas production.

## 2 Major sources of microplastics

Thompson *et al.*,^22^ who investigated maritime plastic contamination in the United Kingdom, were the first to use the phrase “microplastics” 21 years ago. Microplastics have since attracted interest from governments, non-profit organisations, scientists, and others. According to Scheurer and Bigalke, MPs, or particles lesser than 5 mm, are present in both marine and land-based environments.^23^

Plastic products in the environment gradually fragment into smaller particles due to physical, chemical, and biological processes, forming a size-based continuum. These fragments are classified according to their dimensions. Nanoplastics are the smallest, measuring less than 1 micrometer (µm). Microplastics are particles with a size ranging from 1 µm to less than 5 mm (millimeters) and include particles that are either manufactured at this scale or result from the breakdown of larger plastics. Mesoplastics are medium-sized fragments between 5 mm and 25 millimeters (mm). Macroplastics are larger pieces ranging from 25 mm to 100 mm, while megaplastics are greater than 100 mm in size. These categories help to describe the range of plastic pollution found in soil, air, water, and other environmental media. They might even be carried by water and air currents.^24^

MPs and nanoplastics (NPs) are synthetic polymer particles; however, their size is the primary distinguishing factor that determines their environmental behavior, analytical detection methods, and likely toxicity. Nanoplastics are ultra-fine “products” of microplastics, and their capacity to penetrate deeper into biological systems frequently results in increased hazards. The identification of MPs through Raman or FTIR spectroscopy is a relatively standard process. However, the identification of NPs is difficult due to their low concentrations and interference from environmental organic matter. Nanoflow cytometry and pyrolysis-GC/MS are employed to identify nanoparticles (NPs) in environmental and wastewater samples, despite the fact that they demand specialized instruments.^25^

**Fig. 1:**
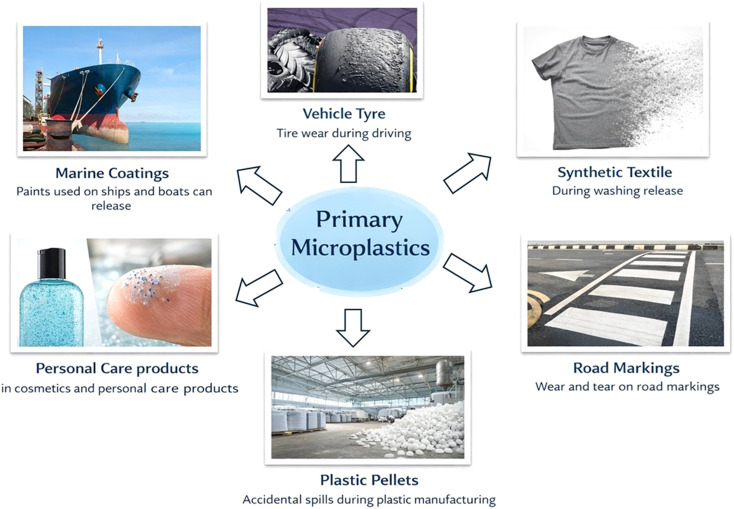
Graphical representation of various primary sources of microplastics. This image was prepared using Microsoft PowerPoint.

**Fig. 2:**
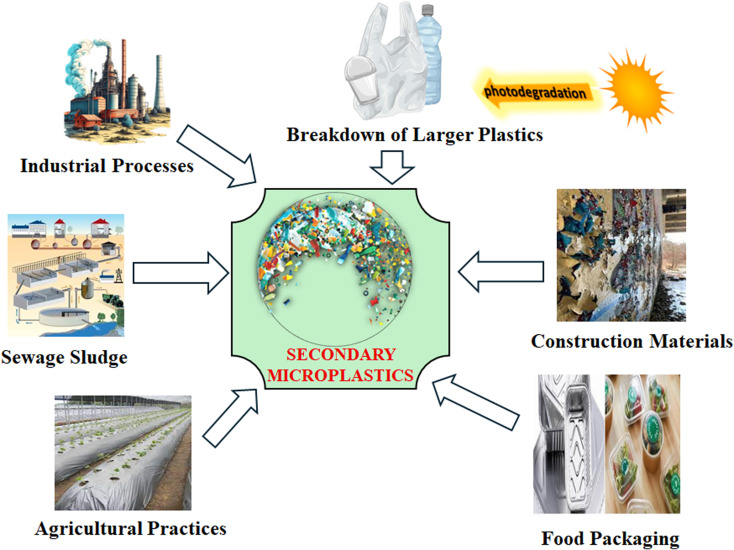
Production of secondary microplastics through different routes. This image was prepared using Microsoft PowerPoint.

Depending on where they are derived, MPs fall into two categories: primary and second-generation. Plastic pieces that have been dumped into rivers and wastewater treatment facilities are known as primary microplastics (Fig. 1). When enormous volumes of plastic waste are broken up and reduced in size by chemical, physical, and biological processes, secondary microplastics are created.^26^ Additionally, tiny broken plastic particles known as microbeads, which range in size from 0.1 mm to 1 mm, are patented components of personal hygiene products. They are used to exfoliate skin on the face, in hand washes, and to increase the viscosity of toothpaste^27^ (Fig. 2).

Microplastics originate mainly from human activities such as tire wear, synthetic textiles, plastic waste degradation, and agricultural runoff. They contaminate soil, water, and air and are transferred between these environments through runoff and atmospheric deposition. In soil, major sources include agricultural mulch, sewage sludge, industrial waste, and fragmented litter from farming and waste management activities. In water bodies, microplastics mainly come from wastewater effluent, land-based runoff, tire particles, industrial pellets, and fishing gear. In the air, they originate from synthetic clothing fibers, tire dust, and industrial emissions. In terrestrial environments, microplastics result primarily from degraded plastic waste, agricultural runoff, and landfill debris.^28^

According to Anderson *et al.*,^29^ MPs exist into five major categories: films, pellets, foam, fibres, and fragments. Microplastics are easily found in six chemically based categories: polyethylene (PE), polystyrene (PS), polypropylene (PP), polyurethane (PU), polyvinyl chloride (PVC), and polyethylene terephthalate (PET). These six prevalent microplastic polymers (PE, PS, PP, PU, PVC, and PET) exhibit substantially varying glass transition temperatures (*T*_g_) as a result of their unique chemical structures, crystallinity, and backbone flexibility. The *T*_g_ is the temperature range within which these polymers transition from a rigid “glassy” state to a soft, rubbery state.^30^

### 2.1 Classification of microplastics

Microplastics are classified into primary and secondary types based on their origin. Primary microplastics are intentionally manufactured in small sizes and are commonly used in cosmetics, detergents, exfoliants, and industrial applications. They are usually uniform in shape, such as pellets, cylinders, or spheres. Examples include microbeads in personal care products, plastic pellets used as raw materials, industrial abrasives, and drug delivery particles.^31,32^ These microplastics enter the environment directly through industrial spills, improper waste disposal, or drainage systems.

Secondary microplastics are formed unintentionally from the breakdown of larger plastic items such as bags, bottles, fishing nets, and clothing. They result from weathering, UV radiation, mechanical forces, and biodegradation, and are typically irregular in shape, appearing as fragments, films, and fibers. Examples include synthetic fibers from laundry, tire wear particles, degraded plastic litter, and paint flakes. These microplastics enter the environment indirectly through litter degradation, urban runoff, and sewage effluent.^26–32^

### 2.2 Land-based sources

It has been demonstrated that single-use products made of polymeric plastics, such as baggage, plastic straws, utensils, coffee containers, and beverage bottles, are a significant source of plastic pollution in the environment.^33^ The excessive usage of single-utilization face masks made of plastic polymers such as nylon and synthetic polypropylene during the COVID-19 pandemic has resulted in a dramatic increase in microplastic waste. The demand for disposable personal protective equipment, including gloves and masks, has increased as a result of the preventive measures implemented by governments and enterprises. It is estimated that approximately 3.4 billion single-use masks or face shields are discarded daily as a result of COVID-19.^34^

Land-based sources of these particles include plastic bags, bottles, personal hygiene items, building supplies, and particles from plastic incinerators, which produce bottom ash that contains microplastics.^35^ This occurs when items such as plastic bottles, bags, and personal care products are burned, leaving behind concentrated microplastic fragments.^32,36^ Industrial processes, particularly those involving aggregates and tiny epoxy granules and sewage effluent are further probable sources of microplastic discharge into the oceans.^37,38^

A number of cosmetics and personal grooming products, in addition to building materials and drugs, are thought to be potential sources of plastic pollution since they may contain microplastics that are either constituents or drug carriers.^39^ Typically, these products include bath gels, toothpaste, face creams, mascaras, cosmetics, sun protection, laundry detergents, face washes, touch soaps, and hand gels.^40^ Polyester, nylon, and acrylics are a few of the chemically produced fibres that have been found to shed from textile products and wind up in aquatic bodies together with wastewater streams.^41^

### 2.3 Marine-based sources (aquatic ecosystems)

Roughly 10% to 20% of the microplastics released into the oceans come from ocean-based sources, including the fishing industry, offshore businesses, marine boats, and coastal tourists.^42,43^ Nylon meshes and plastic monofilament yarn lines are two examples of lost or used fishing tackle that are important producers of microplastics, which can travel in the water at different depths.^44^ This problem continues to grow with the annual disposal of more than 6 lakh tonnes of fishing tackle into the sea.^45^ Also, it is worsen by shipping microplastic debris, which is often released by warships and merchant ships.^46^ Additionally, Calero *et al.* commented that a significant amount of plastic garbage is being discharged into marine ecosystems from offshore businesses such as the petrochemical industry.^47^

Despite being smaller than land-based sources, ocean-based sources play a major role in microplastic pollution, and thus control measures are needed for lowering their contribution. Although 80–90% of microplastics are sourced from land, it is estimated that 10–20% of them are sourced from the marine ecosystem, *i.e.* ocean-based sources, such as fishing nets and cargo.

## 3 Impacts of microplastics

### 3.1 Environmental impact and toxicological impact

The marine environment, waterways, aquifers, wetland ecosystem, the environment, ground soil, and many more ecosystems have all been and continue to be extensively studied for the effects of microplastics.^48–51^ To understand the possible changes in the biological, physical, and chemical attributes of microplastics and their impact on human health, a great deal of information is required. MP particles have been found in a variety of goods meant for people to consume, according to studies.^52,53^ Microplastic particles, particularly nanoplastics, have potent toxicological qualities and can cross numerous biological barriers.^54^ According to Fournier *et al.*, plastic particles are translocated to placental and foetal tissues as a result of maternal lung exposure to nano-polystyrene.^55^ According to Ragusa *et al.*,^56^ MPs are a global issue of the twenty-first century because they have recently been identified inside the human placenta.

MP particles are present in the environment and are a serious issue for various ecological sectors. According to Bergmann *et al.* and Cunningham *et al.*, MPs have been discovered in the deepest parts of the sea,^12,57^ and according to the findings by Napper *et al.*, at the top of the Earth (Mount Everest).^58^ Approximately 80% of MPs come from land, while fewer than 20% come from underwater. One consequence of MPs in the environment is harm to and death of aquatic fish, birds, mammals, and reptiles resulting from the ingestion of aggregated plastics.^59^ The main environmental concerns in recent decades have been terrestrial, marine, and public health; the specific effects of MPs on various environmental sectors are covered here. The toxicity of microplastics is highly variable and depends on a variety of factors, including the specific polymer type, additives such as plasticizers, stabilizers, and pigments, and also their derived structures such as fibers *versus* spheres and size of the polymer. The toxicity levels of all microplastics are not identical. Microplastics with smaller sizes and higher concentrations of added compounds or contaminants that have been adsorbed from the environment are more toxic.^60^

### 3.2 Impact on soil

De Souza Machado *et al.*^61^ claimed that microplastic pollution could pose a serious threat to the land and the surrounding environment, impacting the soil ecosystem chemically and physically by affecting the chemical composition and structure of the soil (dissolving hazardous compounds). The presence of microplastics may also cause changes in the microbiological activity in the soil.^61,62^ Additionally, plastic particles that are injected might spread throughout the terrestrial food chain.^63–65^

Both the upward and downward movement of MPs inside the soil are regulated by a number of elements, such as soil microbes and soil characteristics. Guo *et al.* claimed that MPs change the soil structure when they are incorporated into soil aggregates.^66^ MPs may exist for decades due to the low oxygen and light levels in soils. Therefore, by changing their biophysical surroundings, MPs may have an impact on soil function and biological fitness through interactions. They can, of course, be taken up by plants and passed up the food chain as they accumulate in the soil.

MPs impact the health and performance of growing onions in soil through alterations in nutritional levels, basic tissue framework, root traits, and activity of soil microbes.^67^ Additionally, soil microorganisms like earthworms readily ingest microplastics, which then accumulate in their digestive tracts and are excreted in their casts. Because earthworms form the base of many terrestrial food webs, this bioaccumulation can have long-term ecological consequences for various predatory species and the broader ecosystem.^68^ The influence of MPs on soil organisms was also examined by Lin *et al.*,^69^ who discovered that worm and microarthropod populations declined as MPs increased.

### 3.3 Impact on water

Many human activities cause MPs with diverse chemical compositions from different materials to infiltrate water sources. Some of the most prevalent items are shopping bags, personal grooming goods, and plastic litter such as bottles. According to Cesa *et al.*,^70^ laundry and fishing are two additional primary activities that contribute to the rise in MP discharge in water because tiny, microscopic fibers separate from goods during these processes. Other MPs are intentionally made, such as the tiny plastic beads found in exfoliating cleansers, detergents and cosmetics.

### 3.4 Impacts on human health

It is well recognized that MPs negatively impact human health. The majority of MPs that are ingested through drinking water are probably not absorbed by the digestive system. Consequently, exposure to MPs increases the likelihood of effects including inflammation and irritation in mouth and gut tissues. Polyethylene has been shown to have a cytotoxic effect on T98G and HeLa cells and generate ROS (reactive oxygen species).^71^ Polystyrene also functions as an immunological stimulant that triggers the synthesis of cytokines and chemokines, according to a recent study. It has been demonstrated to be harmful to cells, induce oxidative stress, and impact the fluidity and integrity of membranes. Furthermore, it damages the mitochondrial membrane and stops the ATP-binding cassette (ABC) transporter from functioning in the plasma membrane.

MPs have serious negative effects on humans and the biological environment, including marine life. The marine environment is primarily exposed to plasticisers, which harm its ecology. Additionally, MPs can create composite pollutants that have stronger toxicological effects when they interact with persistent organic matter.^72^ In environmental, agricultural, and aquatic systems, MPs serve as vectors for heavy metals (HMs), adsorbing contaminants such as Pb, Cu, Cd, and Zn as a result of their hydrophobic properties and high surface-area-to-volume ratio. Oxidation, a component of aging processes, increases the number of oxygen-containing functional groups (–OH and –COO), which in turn improves adsorption. This interaction can have synergistic (increased toxicity) or antagonistic (decreased toxicity) effects on organisms, frequently facilitating the bioaccumulation of heavy metals in the food chain.

Finally, the interaction of MPs with heavy metals may increase their toxicity by altering their surface structure and charge in saltwater. Following the consumption of MPs, marine animals may store these harmful compounds, harming the marine habitats and biodiversity.^66^ Furthermore, MPs may have negative health impacts due to their alterations during their movement through the human body.^73^

### 3.5 Quality assurance and quality control in microplastic research

In accordance with recommendations for minimizing environmental contamination, guaranteeing procedural accuracy, and verifying polymer identification, this section describes the minimal necessary quality assurance/quality control (QA/QC) and contamination-control techniques for trustworthy microplastic (MP) research. Any experimental work increases contamination; thus (1) it is necessary to organize and streamline studies to minimize exposure duration. (2) Conduct investigations and maintain samples in recyclable consumables. (3) Avoid the use of aluminium foil to cover samples. (4) Use Milli-Q water when water is required. (5) Experiments should be performed in a biological safety cabinet (BSC) or similar laminar flow cabinet (LAF bench). (6) Frequent cleansing is required to minimize the accumulation and dispersion of laboratory dust, utilizing paper towels and 70% ethanol.^74^

Blanks and controls are essential for monitoring contamination in microplastic analysis. Field blanks detect contamination during sampling, procedural blanks identify laboratory-introduced contamination, and air blanks monitor airborne fibres. Positive controls using standard microplastics are used to assess recovery efficiency and ensure method accuracy. Recovery tests such as spiking should be conducted to assess particle loss and method bias, especially for fibres and particles smaller than 100 µm. Digestion and cleanup procedures must be optimized to remove organic matter without damaging sensitive polymers, avoiding temperatures above 60 °C. Results should include both raw and blank-corrected data, along with the average and standard deviation of particles found in blanks, and a clear statement on whether blank correction was applied.^74^

## 4 Degradation of microplastics

Microplastic (MP) degradation is the process of reducing the size of plastic particles (<5 mm) through physical, chemical, and biological mechanisms. Four main processes lead to the breakdown of microplastics: (1) physical degradation, which breaks polymers into smaller particles due to mechanical forces such action of waves, wear and tear, and cycles of freezing and thawing. Plastics are more vulnerable to additional deterioration as a result of this process, which increases their surface area. (2) Oxidation and hydrolysis reactions are the primary mechanisms by which polymer chains are degraded in chemical degradation. These reactions introduce oxygen-containing functional groups, such as carboxyl, hydroxyl, and carbonyl groups, into the polymer chains. The material is rendered more fragile and susceptible to further fragmentation or eventual biological degradation as a result of these modifications. These reactions are frequently facilitated by environmental factors such as pH, temperature and chemical exposure. (3) Photodegradation is a subtype of chemical degradation, where UV radiation triggers the oxidative breakdown of polymers, leading to fragmentation. Nevertheless, this process is frequently incomplete and results in the formation of smaller microplastic residues. (4) Biodegradation relies on microbial enzymatic activity to metabolize plastics into simpler chemicals. However, this process is extremely sensitive to the ambient conditions (oxygen availability and temperature) and the chemical makeup of the polymer.^75^

While photodegradation and chemical degradation contribute to plastic fragmentation, they seldom result in complete mineralization, indicating that microplastic residues persist in the environment. The term “additive leaching” denotes the discharge of incorporated chemicals, such as plasticizers, *e.g.* phthalates, flame-resistant substances, and chemical stabilizers from the plastic framework into the atmosphere around it. This process does not entail the degradation of the polymer backbone, in contrast to fragmentation or degradation. Rather, it is associated with the absence of non-polymer additives and can occur even when the plastic is physically and chemically unaltered. The following section elaborately describes the various degradation methods.^76^

The natural bio-degradation of plastic materials gradually breaks them down into microplastics (particles smaller than 5 mm) and nanoplastics (smaller than 1 µm) over periods ranging from a few months to several centuries, depending on the environmental conditions. Although the complete biodegradation of conventional plastics may take 450 to more than 1000 years, their fragmentation into secondary microplastics begins much earlier through processes such as photodegradation caused by ultraviolet radiation and mechanical stress.^77^

The degradation timelines of common plastic items vary considerably. Plastic bags typically degrade in about 20 years, while Styrofoam coffee cups may take around 50 years. Plastic straws can persist for up to 200 years, while six-pack plastic rings may take approximately 400 years to break down. Plastic bottles and cups generally require between 450 and 1000 years for degradation, whereas fishing lines can persist for nearly 600 years in the environment.^78^

In general, microplastics with a low molecular weight (LMW) are more easily degraded than those with a high molecular weight (HMW). A lower molecular weight indicates that the polymer chains are shorter, which results in higher mobility, increased accessibility for microorganisms or chemical agents to initiate degradation, and lower tensile strength. In contrast, high molecular weight polymers are more resistant to degradation as a result of their high crystallinity and complex, long-chain structures.^79^

### 4.1 Biodegradation of microplastics

Microplastic bio-degradation is the process by which microbes break down and transform parts of plastic into readily available energy sources by changing the structural group framework, molecular mass, stretching property, and other properties associated with polymers. Environmental variables, MPs characteristics, and microorganisms all influence the bioconversion of MPs. They undergo degradation through two primary processes: oxidation mechanisms and biological breakdown. The enzymatic activity of microorganisms contributes to the biological breakdown of MPs, causing a variety of structural alterations in the micro-plastic polymers. Oxidation mechanisms include photochemical reactions, oxidation, galvanic oxidation, and decomposition of MPs through a variety of ways when exposed to light and reactive oxygen species (ROS). Variable factors such as pH, humidity and temperature are also important parameters for the biological breakdown of organic pollutants.^80–82^

*Pseudomonas* and *Bacillus* are crucial microbes for the breakdown of MPs. *Pseudomonas* proved to be the most effective of 15 bacterial strains obtained from the seashore line that could break down high-density polyethylene (HDPE).^83^ The ability of *Pseudomonas* to degrade MPs seems to be influenced by its potent hydrophobicity, which makes it easier for it to cling to the polymer and form biofilms that aid in its breakdown. The release of additional polysaccharides from cells is essential for quickening the breakdown of low-density polyethylene. According to Shah *et al.*, different plastic types have different degrading effects.^81^

Biofilms have a significant impact on degradation, which is dependent on the establishment of nutritional conditions. In a study, the decrease in the amount of carbohydrates and ammonium sulphates suggested that the hydrophobic nature of the surface of the microorganism had changed.^84^ Accordingly, degradation was positively connected with the enhanced water-repellent nature of the cell boundary, which facilitated effective biofilm development in a polyethylene succinate film.^84^ However, the presence of sea sediments, which are high in naturally occurring carbon, decreased biofilm formation and stopped the disintegration of polyethylene.^85^ Thus, *Pseudomonas* breaks down synthetic plastics in response to environmental and nutritional factors that promote the formation of biofilms on plastic polymers.^86^

### 4.2 Method of bio-splitting

According to Kjeldsen *et al.*,^87^ bio-splitting refers to the process by which microplastic polymers are biologically split or fragmented with the aid of enzymes, which is also referred to as bio-fragmentation. At this instance, the polymeric structure of plastics is hydrolyzed by enzymes such as oxidoreductase and hydrolase. However, several oxidation reactions that produce free radicals are catalyzed by other enzymes. When the polymer oxidizes, free radicals and functional groups such as hydroxy and carbonyl compounds are produced. The enzymatic breakdown of microplastics is subject to a broad range of variations, which are influenced by the material and environmental conditions. For specialized, optimized industrial conditions, the reaction may take as little as a few days, while the biodegradation of specific polymers may take several weeks or months. In natural environments, the process is typically slower, with some studies demonstrating a 7–63% reduction over several weeks, depending on the enzyme type.^80–83,88,89^

### 4.3 Mineral formation or integration

During this stage, microbes absorb monomers to create the microbe population, carbon dioxide, and methane.^83^ The microbial cells use these monomers for growth and they provide carbon atoms for the production of energy.^90^ However, some plastic monomers are challenging to absorb because of the selective permeability of plasma membranes. Through the process of biotransformation, microbial cells make use of non-assimilated monomers. Microorganisms utilize unbound monomers from polymers *via* a process termed modification, wherein the enzymes secreted by these microbes transform chemical substances into final products that can be absorbed by other microorganisms or similar species.^89^

The appropriate physio-chemical characteristics of biodegradable polymers, such as their high flexibility, abundant functional groups, and relatively low molecular weight, make them more amenable to pretreatment than conventional plastics. Pretreatment of plastics using a range of physical and chemical agents can change their morphological and structural properties, including lowering their molecular weight, breaking chemical bonds, forming surface cracks, and adding functional groups.^90^ It is important to ascertain how various pretreatments affect the biodegradation of plastics and MPs, as this can increase the degradation percentage even further. High-temperature pretreatment, photooxidation catalysis, and microbial enzyme catalysis may facilitate the effective biodegradation of plastic polymers.^91^

### 4.4 Bio-degradation of matter

Enzymes break down microplastic polymers into their constituent monomers during this stage of breakdown. Both inside and outside the plastic material, the microplastic polymers are disrupted.^82^ The types of enzymes that microorganisms secrete, such as lipases, proteases, and urease, are necessary for the enzymatic breakdown of microplastics.^84^

Currently, considerable research focuses on how microorganisms such as bacteria, fungi and algae break down aquatic microplastics, along with understanding the molecular mechanisms underlying their partial chemical breakdown.^92–95^ Through enrichment and culture, bacteria including *Acinetobacter*, *Bacillus*, *Pseudomonas*, and *Klebsiella* have been progressively isolated from wastewater, sludge, and plastic waste. Additionally, numerous genes and enzymes implicated in the breakdown of plastic have been consistently discovered.^87–90^

The primary fungi that can break down (micro)plastics are *Xanthosporea pinnatifida*, *Aspergillus*, *Dendrosporum*, *Fusarium*, and *Penicillium*. Their mycelial structures maximise the breakdown of microplastics by efficiently penetrating the surface of the polymer material and reaching its interior. Furthermore, fungi have the ability to produce a variety of oxidative enzymes and biosurfactants, which show exceptional efficacy in breaking down microplastics.^96^ Gao *et al.*^97^ isolated a strain of aquatic fungus. The alternative species FB1 demonstrated a degradation rate as high as 95% for polyethylene film within 120 days. Diglycolamine was the principal breakdown product, and laccase and peroxidase were the predominant degradation enzymes. Thus, there is a lot of room for research into identifying fungi that can break down microplastics from the environment, and the precise molecular pathways underlying this process are still unclear.

Microalgae offer tremendous application prospects since they are more adapted to the ocean than both fungi and bacteria, and they can perform photochemical processes and self-supporting nourishment for degrading marine microplastic contamination.^98^ Cyanobacteria, algal blooms, and diatoms, in particular, are the primary microalgae that have been shown to be able to break down microplastics.^99^ Microalgae that stick to plastic surfaces produce extracellular polysaccharides and ligninolytic enzymes, which are essential for the breakdown of plastic and help lower the activation energy required to break the chemical bonds of polymers.^100^

For a long time, environmental engineering, ecology, and environmental studies have considered MP pollution. Additionally, the breakdown of MPs by microorganisms provides a more humane means of combating microplastic contamination. The characteristics of microplastics affect the primary difficulties that arise when bacteria break down microplastics. The enzymatic reactions by microorganisms cause plastic to degrade. However, despite the numerous publications, it is still unclear which approach is best for the degradation of plastics, in addition to the microbes linked to MP degrading processes. The breakdown of bigger plastic particles into smaller ones is aided by various bacterial, fungal, and algal strains. Enzymes, both intracellular and extracellular, are also crucial for the breakdown of microplastics. Another method involves the degradation of microplastics *via* the mechanisms of oxidation, photocatalysis, and photochemical and electrochemical degradation.^101^

### 4.5 Degradation by oxygenation mechanism

The method reported by Du *et al.* works well for breaking down persistent contaminants. The production of reactive oxygen species is the basis for the breakdown of organic contaminants in this process.^102^ Reactive oxygen species directly initiate the decaying process by rupturing the long chain of polymers and completing the deterioration cycle through the production of valuable products.^103^

### 4.6 Degradation by photocatalysis

Using highly renewable solar energy, photocatalytic degradation is an environmentally friendly method for breaking down organic contaminants. The breakdown of semiconductor components in organic pollutants is the basis of this process. Breakdown commences in the semiconductor components when the photon energy substantially exceeds the bandgap energy of the semiconductor materials. In semiconductor materials, electrons from the outermost valence band easily migrate to the conduction band, forming a positive hole in the valence band and creating an electron–hole pair.^102^ Reactive oxygen species are produced when free hydroxyl radicals interact with both electrons and holes, and MP breakdown is directly triggered by the free reactive oxygen species.^104^

### 4.7 Degradation by photochemistry

Organic contaminants can also be broken down by photochemical degradation. Photochemical degradation is significantly influenced by ultraviolet (UV) radiation.^105^ Long-term exposure to UV radiation causes organic contaminants to breakdown, creating free radicals from oxygen and a lengthy polymer chain with a cross-linkage.^106^

### 4.8 Degradation by electrolysis

This approach is based on the anodized and cathodic surface deterioration of pollutants. Anodic degradation leads to passive oxidation *via* reactive oxygen compounds and hydrogen peroxide, as well as direct oxidation by charge transfer on the surface of the anode of impure substances. Oxygen free radicals and the electron Fenton method accomplish cathodic degradation of MPs. MP breakdown is caused by the Fe^+^- generated reactive species.^102^

On comparing the all above-mentioned methods that are employed to degrade microplastics, grinding, heating, and UV exposure are physical methods that are both efficient and effective in reducing particulate size. However, they are energy intensive and frequently result in the production of smaller microplastics. Advanced oxidation and photocatalysis are chemical methods that can completely decompose plastics into simpler compounds. However, they are costly and may result in secondary contamination. Biological methods are environmentally benign, but they are slow and have low efficiency, as they rely on microorganisms and enzymes to degrade plastics. In general, physical methods are rapid, chemical methods are highly effective but expensive, and biological methods are sustainable but slow to adopt.^107^

### 4.9 Degradation by plasma-assisted method

Plasma technology has demonstrated an exceptional performance in the treatment of microplastic waste and small-scale microplastic pollution, as evidenced by recent experimental results. For instance, a study integrated air plasma with electrofluid technology to degrade standard plastics, including polypropylene (PP), polyethylene (PE), and polyvinyl chloride (PVC). This approach has the potential to achieve a degradation efficiency of over 87%. Plasma technology offers a variety of benefits, including the absence of chemical reagents throughout the entire treatment process, which minimizes the risk of secondary pollution at the source. Additionally, its non-thermal properties render it suitable for heat-resistant materials. Finally, its robust compatibility enables it to be combined with other environmentally friendly processes, such as catalytic systems, photochemical reactions, ozone treatment, or ultrasonic technology, to create a multi-pathway degradation strategy that enhances the rate of degradation.^108^

### 4.10 Removal of microplastics by adsorption mechanism

A growing body of research highlights adsorption as an efficient, economical, and versatile method for removing MPs and nanoplastics (NPs) from water and wastewater, often achieving over 90% efficiency. Removal occurs through mechanisms such as hydrophobic and electrostatic interactions, hydrogen bonding, pore filling, π–π stacking, and surface complexation, depending on the adsorbent used. Nanomaterials and bio-based adsorbents show a particularly high performance due to their unique properties. However, their effectiveness depends on environmental conditions, and challenges related to large-scale application and potential risks remain. Despite these limitations, adsorption-based materials offer promising solutions for reducing microplastic contamination in water systems.^109^

### 4.11 Removal of microplastics by metal–organic frameworks

Metal–organic frameworks (MOFs) are highly proficient in the removal of microplastics from water samples, attaining adsorption and photocatalytic degradation rates of 70–99.9%. Their extensive surface area facilitates capture through electrostatic interactions, hydrogen bonding, and customized pores, while light-induced radicals contribute to the degradation of plastic surfaces. MOFs are also employed in composite membranes to enhance filtration and minimize contamination, while waste PET can be recycled to generate functional MOFs. Nevertheless, the large-scale implementation of these compounds is restricted by their high synthesis costs and long-term stability challenges.^110^

In the studies carried out by Barari Fateme *et al.*,^111^ they focused on the important role of MOFs in controlling microplastic pollution, particularly polystyrene (PS), which is widely present in the environment. The adsorption efficiency is influenced by microplastic concentration, particle size, contact time, and pH. About 32% of studies focused on PS at concentrations of 10–100 mg L^−1^, while most experiments used 100–1000 mg L^−1^. Nearly half of the studies examined contact times of over 200 min, indicating improved interaction with longer exposure, and 36% were conducted at pH 3–6, showing strong pH dependence. The main adsorption mechanisms include electrostatic attraction, acid–base interactions, and π–π interactions, with the pseudo-first-order and Freundlich models best describing the process. Additionally, the regenerability of MOFs supports their potential as sustainable and cost-effective materials for microplastic removal.^112^

## 5 Analytical methods for the identification of microplastics

### 5.1 Direct visual and microscopy methods

Direct visual techniques, optical microscopy findings, and electron microscopy analysis are visual inspection methods that can be used to choose and categorize microplastics based on the size and colour of the objects visualized under an optical microscope or with the bare eye. In these counting microscopy techniques, particles are counted directly and they are capable of recognizing particles in the millimetre (mm) range. Their advantages include the ease of identifying samples with a substantial quantity of larger-size microplastics, providing a rapid and cost-effective overview of microplastic richness. However, restrictions necessitate a combination of identification methods, as the composition of the materials is not known.^113–115^

Polarised light microscopy was used by Mossotti *et al.* to successfully identify polyethylene (PE) particles in toxicity testing.^116^ The crystal structure of the plastic may have an impact on the transmission of polarized light, which can be studied.^117^ However, to allow sufficient polarized light to pass through, small microplastic particles are necessary. Also, this technique is not applicable to opaque microplastic samples. The study of nanoplastics could benefit greatly from the use of dynamic light scattering. In a study employing dynamic light scattering, the authors discovered that the solar photochemical degradation process may convert second-hand microplastics into new nano-form plastics.^118^

It is possible to quickly and automatically study the soil dispersion and sizes of sedimentary particles accurately using laser diffraction particle size analysis.^119^ Blott *et al.* claimed that particles ranging in size from 0.04 micrometer to 2000 micrometer can be studied using this method; however, the results of the study could be distorted by several pollutants present in surrounding samples.^120^ While this method is not commonly employed for determining the size dispersion of microplastic particles, it is poised to become increasingly significant in this field as technological advancements continue.

Furthermore, MPs cannot be reliably distinguished from the vast array of other organic and inorganic substances including fibres from cellulose and starch waste by visual inspection. It has been observed that various parameters, such as the properties of the sample matrix, the quality of microscopy, and individual characteristics, significantly influence the process of visual inspection and the determination of microparticles. Additionally, the size constraint of some small-sized MPs is a disadvantage of the optical counting method. Visual inspection resulted in error rates as high as 70% with a decrease in MP size, indicating an increase in the number of errors.^113^

### 5.2 Analytical spectroscopic methods

The spectrum analysis technique provides more accurate information than visual identification. The polymer types in MP particles with the smallest particle sizes of 10 mm and 1 mm have been identified through Fourier-transform infrared spectroscopy and Raman spectral analysis.^121–124^ Raman spectroscopy (RS) serves as an essential method for determining the chemical formulation of MPs across various water sources, including freshwater, groundwater, drinking water, ocean water, and wastewater. According to Raman spectroscopy, the common MPs identified in water sources are polystyrene, polyethylene terephthalate, polyethylene, and polypropylene.

However, due to the inadequate diffraction resolution of the instruments, these two approaches have poor spatial resolution. Furthermore, the slow process of Raman scattering reduces the strength of the signal, presenting a disadvantage. Consequently, the theoretical spatial resolutions for Raman spectroscopy and FTIR are 1 µm and 20 µm, respectively.^125^ Additionally, the size, colour, density, and additives (such colorants and plasticizers) of MP particles vary, making their detection much more difficult. This indicates that without further modifying the aforementioned techniques, microplastics cannot be detected efficiently.

Surface-enhanced Raman spectroscopy (SERS) is a method for increasing the sensitivity of Raman spectroscopy.^126^ Many chemical compounds, including pharmaceuticals, contaminants, biomolecules, explosives, and microscopic plastic particles, can be detected in low quantities using this reliable technique.^127–130^ A crucial factor influencing enhanced spectroscopy amplification is the either dynamic or static aggregation of nanoparticles within the dispersion. As an output, a group of microparticles may have plasmonic characteristics distinct from those of single MPs, as well as higher plasmonic resonance wavelengths, hotspots, and enhancement efficiencies.

Xu *et al.* employed the commercially available substrate Klarite in the SERS detection of polystyrene and polymethyl methacrylate microplastics with a size as small as 360 nm.^131^ Intense surface-enhanced Raman spectroscopy signals were obtained from polystyrene nanoplastics trapped inside Ag nanowire network structures fabricated and coated on a renewable cellulose matrix in the study by Jeon and colleagues.^132^ For 46 nm AuNP substrates, Caldwell *et al.* obtained detection limits of 20 microgram millilitre^−1^ for the 33 nm PS, 15 microgram millilitre^−1^ for 62 nm PET, and 10 microgram millilitre^−1^ for 161 nm PS.^133^

A successive milling technique for creating PET particles in the nanometre range was created by the same team.^134^ As polystyrene (PS) nanospheres move along unique golden solid tiny pores, they may also be identified by SERS.^135^ Alternatively, according to the latest report by Lv *et al.*, SERS allows for the detection of 100 nm-sized polystyrene plastics (at a concentration as low as 40 µg mL^−1^), differentiating among microplastic particle types such as PE and PP.^136^ Silver nanoparticles (AgNPs) were aggregated with salts to produce this improvement. A study demonstrated for the first time that SERS mapping could be used to produce a sequence of Raman spectra presenting chemical information when polystyrene nanoplastics are surrounded by aggregated silver nanoparticles on an enhanced Raman substrate.^137^

In a study, indiscernible plastic constituents as small as about 50 nm spiked in sample water could be detected using the suggested technique. Hu and colleagues^138^ developed an SERS method based on a silver colloid for identifying nanosized polystyrene plastics down to 50 nm in size. Recently, Lee and Fang devised a technique for detecting 600 nm polystyrene particles using gold nanoparticles as SERS substrates.^139^

Methods based on mass spectrometry are also employed to examine the polymer type in MPs. MPs need to be burned or digested for the analysis of liquid or gas samples in mass spectrometry. Consequently, this method can only determine the type of polymer and the quantity of MPs, not the dimensions or morphology of the MP particles. Advances in isotope tagging technology have led to the development of a unique mass spectral analysis for determining the quantity of microplastics by adopting the ICP-mass spectrometry technique. The detection and sizing of nanoparticles have also been made possible using the above-mentioned technique.^140^ This approach is extensively utilized for analysing metal-based nanoparticles in environmental samples.^141–143^

For instance, because of its comparatively small limit of detection of 8.4105 particles per L, single-particle ICP-MS has been used to evaluate the dimensions and total concentration of simulated gold-coated microplastics at the submicrometer scale. Nevertheless, its usage necessitated extensive sample preparation owing to its dependence on the non-direct assessment of the gold coating .^144,145^ By tracking ^13^C as the process progresses, single-particle ICP-MS has been employed to evaluate the dimensions of particles and amount of experimental plastic microparticles.^146,147^

Pyrolysis GC-MS is an effective and sensitive method for the characterization and measurement of the mass of various polymeric material types and their organic additives.^110,148,149^ It has been demonstrated that pyrolysis GC-MS works effectively with µ-ATR-FTIR spectroscopy to detect microplastics in environmental samples.^150^ However, it has a number of disadvantages. For matrix-rich materials, thorough sample clean-up is necessary due to the extremely small pyrolysis capsule size of 1.5 millimetre and 0.5 milligram, making it inappropriate for bulk analysis. It is also susceptible to obstructions or contamination. Dumichen *et al.*^151^ developed a thermal extraction desorption gas chromatography-mass spectrometry technique for the detection of microplastics to address these challenges.

Liquid chromatography-tandem mass spectrometry was successfully employed for the effective detection and quantification of MPs.^152–155^ However, this method is unfavourable since it requires depolymerising macromolecules prior to examination. The analytical method provides data on the total amount and volume of monomer molecules produced during degradation, in relation to the number, shape, colour and particle size of microplastics.

Majewsky *et al.*^156^ used differential scanning calorimetry (DSC) combined with thermogravimetric analysis (TGA) to analyse microplastics in wastewater, and according to the analysis report, only the materials polyethylene and polypropylene were readily easily distinguished. Without sample preparation, using TGA and mass spectrometry, David *et al.*^157^ attempted to quantitatively analyze polyethylene terephthalate (PET) in soil samples. Despite its success, this technique still needs to be improved and is limited to PET analysis alone. Chromatography and thermogravimetric analysis necessitate more rapid and potential methods for the quantitative identification of microplastics in soils and other complex environments.^158^ However, the destructive character of these processes precludes additional research, and the fact that the amount, structural form, and size parameters of the particles are unknown contributes to their limitations.

Castaneda *et al.*^159^ stated that the formation of polyethylene and other important microscopic particles commonly found in the environment are often discovered using differential scanning calorimetry (DSC). Since all plastic items have distinct qualities, DSC can be used to differentiate between various polymer types.^160^ However, there are restrictions since the peaks overlap when DSC detects microplastics with identical melting points.^161^ PP and PE are two of the main types of microplastics that can be identified by DSC, although it cannot identify all microplastics.

Addressing the issue of microplastics can be approached through a simple staining method. Nonetheless, following numerous endeavors to utilize alternative dyes, the fluorescent dye 9-diethylamino-5H-benzophenoxazine-5-one demonstrates remarkable efficacy in selectively staining extremely water-resistant microplastics. Another option is Nile red. Lipids that are physiologically neutral are commonly stained with this dye, but only in a hydrophobic environment. The Nile red staining method presents two notable advantages: brief staining durations ranging from 10 to 30 min and impressive recovery efficiencies that can reach up to 96%. An important drawback of staining materials with Nile red is the possibility of *in situ* staining of multiple organic or chemical molecules. Accordingly, a meticulous cleaning process is essential for effectively preparing samples for staining with Nile red dye. However, the extensive range of plastic densities constrains the efficacy of this approach. Alternatively, Nile red staining is an effective first step for locating hidden microplastics before a more in-depth spectroscopic analysis.^162,163^

Another method for assessing microplastics reported by Zhang *et al.* is the study of near-infrared (NIR) spectra^164^ by employing NIR spectroscopy and performing NIR analysis over a wide spectral range from 4000 to 15 000 cm^−1^. According to Paul *et al.*,^165^ NIR analysis can be used to identify the type of sample rather than its quantity. It is also simple to filter and evaluate vast amounts of data from plastic sample sets.

The detection of MPs in water through impedance spectroscopy is a promising, label-free technique that offers high-throughput, *in situ*, and cost-effective monitoring, specifically employing electrical impedance spectroscopy (EIS) and impedance cytometry.^166^ Nevertheless, this method is still in the early stages of development and encounters numerous substantial obstacles in the identification, differentiation, and quantification of particles within intricate environmental samples. At present, its constraints include the inability to differentiate plastics from other environmental detritus, the challenge of coping with high conductivity, and its sensitivity to small or biological particles.

For a variety of polymers, an open Vis-NIR spectral database can be used to identify common microplastics such as PET, LDPE, and PVC. This approach determines the reflectance at various wavelengths by quantifying the reflected light from the surface of the sample within the 350–2500 nm spectral range. Nonetheless, owing to its dependence on optical detection, there remains a possibility that biological particles could be erroneously identified as plastic materials, necessitating human decision-making.

To discern microplastics within diverse surroundings, an array of analytical techniques can be employed (Table 1). The diminutive nature of microplastics renders their detection increasingly challenging. Analysis at sub-micron levels is increasingly crucial for evaluating the adverse effects of microplastics on both environmental systems and human well-being. Innovative dyeing techniques, nanotechnology, and analytical approaches should all be further developed in studies to detect and remove microplastics from environmental samples.

**Table 1:** Analytical techniques employed for the detection of microplastics

S. No.	Technique	Basis of operations	Advantages	Disadvantages	Ref.
1	FTIR spectroscopy	This method uses the Fourier-pair relationship between a spectrum and its interferogram. Infrared light passes through a Michelson interferometer and is absorbed by molecular bonds at specific frequencies	Safe, non-destructive, and highly specific to certain polymer types	Only functional groups are detected, not individual molecules. Limited by particle size and potential substrate contamination	167 and 168
2	Raman spectroscopy	Raman spectroscopy uses a laser to induce scattering and generate a fingerprint spectrum, enabling qualitative analysis by comparing chemical structures to reference samples	Raman spectroscopy offers high depth resolution, enabling accurate identification of microplastics even at very small sizes	Raman spectroscopy can be affected by dyes, additives, and fluorescence, leading to signal interference, longer analysis times, and difficult identification	169–171
3	Inductively coupled plasma mass spectrometry (ICP-MS)	ICP uses high-temperature argon plasma to atomize and ionize microplastic samples, which are then analyzed by mass spectrometry. Specific *m*/*z* values, like 13C^+^, allow the detection and quantification of individual microplastic particles	ICP-MS detects microplastics at ultra-low concentrations, providing particle count, size distribution, and elemental composition. It enables source identification through isotope analysis and offers faster results than microscopy	Detection of carbon-based microplastics can be hindered by other carbon sources like organic matter and microbes. Interfering elements may affect signals, requiring calibration and often confirmation by FTIR or Raman methods	146 and 172
4	SEM-EDX (scanning electron microscopy with energy dispersive X-ray spectroscopy)	SEM-EDX detects microplastics in water, soil, and tissues by analyzing X-rays to determine elemental composition. It also reveals contaminant adsorption, aiding toxicity assessment	SEM provides detailed images of microplastic morphology, while EDX identifies elemental composition, aiding polymer identification. Together, they offer a powerful tool for comprehensive microplastic analysis across various environmental samples	SEM-EDX requires time-consuming sampling, and its element detection limits can affect trace or tiny microplastic analysis. Similar elemental compositions may hinder accurate differentiation	173 and 174
5	Pyrolysis GC-MS	Microplastics are thermally degraded into volatile compounds, which are separated by chromatography and identified by mass spectrometry based on their mass-to-charge ratio, enabling polymer type identification	Requires only a small sample and enables microplastic identification in salt, soil, sediment, and water with minimal interference from additives or MP properties like color, size, and shape	Identifying all polymer types is challenging due to matrix effects and thermal degradation products; internal standards are essential for accurate analysis	175 and 176

### 5.3 Electrochemical detection of microplastics

Electrochemical detection is dependent on the electrically insulating properties of the majority of synthetic polymers to produce measurable signals, such as impedance changes or current obstructions. Electron transfer, physical adsorption, frequency of collisions at the electrode-solution interface, and particle size, surface charge, and functional groups that are unique to polymers directly influence the electrochemical signals in the detection of MPs.^177^

The distinctive electrical properties demonstrated by MPs and various particles within an electrical field have facilitated the extensive application of electrochemical sensors in the detection of contaminants in the environment. Particularly, the rise in specialized electrochemical sensors for MPs is noteworthy, which have benefits such as quick response times, easy operation, portability, and cost-effectiveness in comparison to other approaches. Moreover, electrochemical methods work better than traditional ones since they allow for the easy on-site assessment of different types of samples and do not require MP isolation or purification beforehand.

Shimizu *et al.* utilized particle impact electrochemistry, which enabled the successful detection of polyethylene circular microbeads with diameters ranging from 1 to 22 micrometers.^178^ Particle-electrode impact is a widely used method for analysing particles dispersed in a solution medium. During chronoamperometry testing, a rapid current response was seen when particles collided with a microwire electrode made from carbon fibre. An undivided three-electrode configuration was used for the electrochemical analysis, and a certain voltage was applied to achieve the desired reaction. The transitory current response, sometimes referred to as a “spike,” was caused by particles colliding with the electrode and the collision factor, which led to an alteration in the observed signal and helped the sensors to detect particles directly. This spike was analyzed for the presence of MPs.

To detect polyethylene-based microplastics, Colson and Michel^179^ employed flow-based cytometry in combination with an impedance spectroscopy-based sensor. The initial step toward the development of a high-throughput, *in situ* sensor (impedance signatures) for the quantification of microplastics in freshwater bodies is the use of impedance spectroscopy in the laboratory for the detection of microplastics in tap water due to its effectiveness.

A flow cytometry component was employed that relied on the relationship between particle volume and the variation in the real part of impedance at low frequencies. The path utilized by the microplastics across the electrodes caused an impedance change that was exactly proportional to the particle volume and especially visible at low frequencies. By establishing a linear correlation between resistance change and particle size through the relationship between particle size diameter and the cubic root of the actual resistance change, the sensor was able to successfully determine the amount and size of microplastic beads from 210 to 1200 µm and polyethylene microplastics from 212 to 1000 µm.

Microplastics were investigated by Gongi *et al.*^180^ at varying concentrations of extracellular polymetric substances (EPS) between 10^−11^ and 10^−5^ M. A three-electrode assembly was utilized in this experiment, in which the working electrode was a gold electrode treated with EPS. Platinum served as the counter electrode, whereas a saturated calomel electrode served as the reference electrode. Four different types of microplastics of different sizes could be detected, including polystyrene, which had a size of 0.1 µm, polymethyl acrylate, with a size of 10 µm, nylon polyamide, with a size of 50 µm, and low-density polyethylene, with a size of 1 mm, among the found microplastics. Sensors can detect microplastics by measuring the variations in electric signals generated through the kinetic binding of each type of microplastic to the electrode surface. Cyanobacterial extracellular polymeric substances (EPS) function as bioreceptors with a strong affinity for various types of microplastics, thereby generating a consistent and dependable signal. The EPS resistance (*R*_m_) increases as the concentration of MPs increases. However, interference investigations have not been documented.

**Fig. 3:**
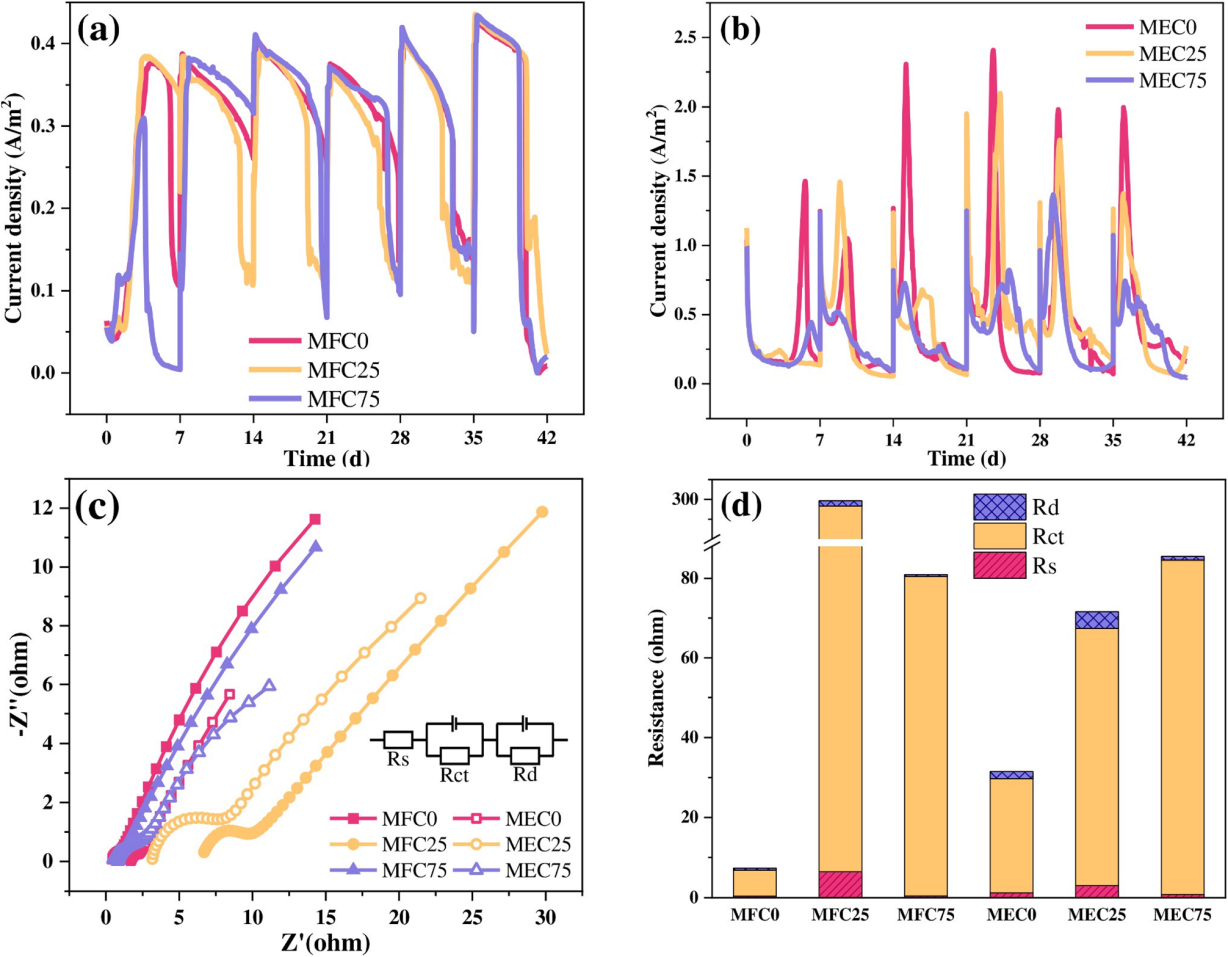
Performance and electrochemical activity of MESs. (a) Current density output in MFCs; (b) current density response in MECs; and (c) Nyquist plots and (d) internal resistance analyzed by fitting to the equivalent circuit ((c), inset) for the anode biofilm *via* EIS, respectively. This image was adapted from the reference under a CC-BY license.^181^

To identify polyethylene microplastics (PE-MPs) in wastewater, Wang *et al.*^181^ suggested employing electroactive biofilms in microbial electrochemical systems (MES). The setup consisted of an Ag/AgCl reference electrode, titanium mesh cathode, and carbon fiber brush anode. MP exposure changed the characteristics of the biofilm and increased the internal resistance, primarily because of the greater charge transfer resistance (*R*_ct_), as demonstrated by impedance spectroscopy. This increase was connected to both enhanced cell death and MP binding. MECs exhibit promise for future wastewater cleanup applications, MP quantification, and type/size discrimination. They examined the potential effects of PE microplastics on the microbiology and electrochemistry of exoelectrogenic biofilms in microbial electrolysis cells (MECs) and microbial fuel cells (MFCs). They showed how PE-MP affected the electrogenic bacteria in MFCs and MECs. It was discovered that the current density decreased as the PE-MP concentration increased in MECs but did not significantly change with the presence of PE-MP in MFCs. Fig. 3(a–d) shows the performance and electrochemical activity of MESs and their current density responses along with Nyquist plots.

In a recent study, Du *et al.*^182^ suggested a novel sensor for polymeric styrene microplastics, which were detected at different particle sizes ranging from 0.08 to 20 µm and concentrations from 0.01 to 25 mg L^−1^ using graphene electrodes and impedance spectroscopy. The greatest *R*^2^ value of 0.9914 was achieved for PS, specifically for particles with a size of 1 µm, showing a strong linear association. These results demonstrate the accuracy and reliability of the sensor in measuring PS amount and particle size in actual samples. Electrochemical impedance spectroscopy (EIS)-based graphene electrode sensors operate by detecting fluctuations in electrical impedance (resistance and capacitance) at the electrode/electrolyte interface as a result of analyte adsorption or binding. The tunable electronic structure, rapid electron transfer capability, and high specific surface area of graphene make it a highly sensitive transducer for the detection of microplastics, frequently at low concentrations.

**Fig. 4:**
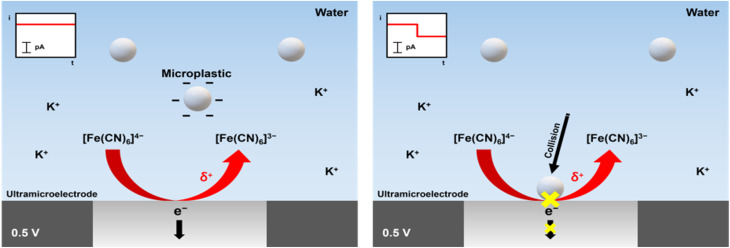
Schematic of the current change caused by microplastics colliding with the electrode. This image was adapted from the reference under a CC-BY licence.^183^

**Fig. 5:**
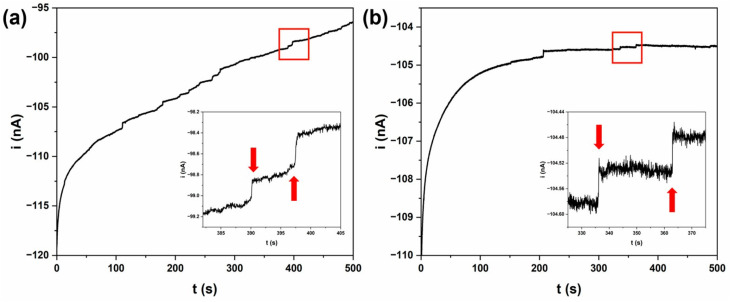
Amperometric *i*–*t* curves of solution containing 100 mM potassium ferrocyanide and microplastics produced by grinding (a) disposable storage containers (PS) and (b) plastic cups (PP), using a 10 µm Pt UME at +0.5 V (*vs.* Ag/AgCl). The insets offer a magnified view of the area boxed in red; arrows highlight the discrete current steps resulting from microplastic collisions. This image was adapted from the reference under a CC-BY licence.^183^

Changhu Lee *et al.*^183^ suggested a single-entity electrochemistry approach for detecting microplastics in water, emphasizing the interaction between the electrode surface and microplastics in aqueous solution. Fig. 4 shows the current change brought on by microplastics striking the electrode surface and how to detect real microplastics using an ultramicroelectrode (UME), which is made by hand-grinding throwaway plastic containers to resemble the microplastics that are found in aquatic ecosystems naturally. The amperometric *i*–*t* curves of a solution containing potassium ferrocyanide and microplastics from grinding plastic cups made with polypropylene (PP) and disposable storage containers made with polystyrene (PS) with a Pt UME are displayed in Fig. 5a and b. The arrows point to the step current produced by microplastic impacts, and the inset photos offer a closer look at the area delineated in the red box.

Utilizing the surface charge characteristics of polystyrene and polypropylene microplastics, they used the migration phenomenon brought on by the ongoing oxidation of potassium ferrocyanide to draw these particles to the electrode surface. The sudden shift in the steady-state current upon the microplastic contacting the UME surface indicated that the microplastic detection was successful.

To detect PVC microplastics in aquatic environments, Surucu, Ozge^184^ demonstrated a new type of chemical sensor. In his work, a composite of graphene oxide (GO), multi-walled carbon nanotubes (MWCNTs), and silver nanoparticles (AgNPs) on a gold electrode was used to create a novel electrochemical sensor. With a detection range of 1.00–5.00 mg mL^−1^ and a limit of detection (LOD) of 0.79 mg mL^−1^, the modified electrode identified PVC microplastics in freshwater and saltwater at −0.30 V *vs.* Ag/AgCl, as described by SEM-EDX. Heavy metals were also detected in the samples by ICP-MS analysis, demonstrating the long-term prevalence of PVC in marine habitats.

The latest research focuses on electrochemical sensors^185^ that detect microplastics by measuring changes in electrical signals brought on by interactions between microplastics and the sensor surface using techniques such as voltammetry and impedance spectroscopy. Particle size, charge, and functional groups unique to a polymer can all affect these signals. Low-concentration detection is made possible by the enhancement of sensitivity, conductivity, and surface area provided by sensor materials such metal oxides, graphene, carbon nanotubes, and nanocomposites. Selectivity is further enhanced by modified electrodes with dye tags or chemical recognition units (Table 2). The requirement for resilient designs is highlighted by the fact that performance varies with water matrix complexity.

**Table 2:** Detection of microplastics by different electrochemical sensors

Electrode material	MP type	Study	Linear range	Advantages	Detection limit	Ref.
Bi_2_O_2_S/CuBi_2_O_4_ photocathode–photoanode	PS microplastics	Dual photoelectrode PEC sensor	0.5–1000 µg mL^−1^	High photocurrent response	0.09 µg mL^−1^	186
Carbon SPE + FITC-tagged PS-binding peptide	PS MPs	EIS + peptide sensor	50 ppb–20 ppm	Strong EIS change per ppb-level PS	50 ppb (pure/tap); 400 ppb (saline)	187
ITO + chitosan-MgO nanosheets	HMT additive (proxy)	DPV nanocomposite sensor	0.5–4.0 µM	Sensitivity of 12.9 µA µM^−1^ cm^−2^	0.03 µM	188
Carbon fiber UME (∼10 µm)	PS and PP MPs	Ultramicroelectrode collision method	Particle count proportional	Single-particle current step detection	Approx. 10^3^–10^4^ particles per mL	183
Waste-derived graphene	PS (also PA, PMA, and PE)	Graphene EIS sensor	0.01–25 mg L^−1^	High impedance change per mg L^−1^	0.01 mg L^−1^	182
Gold + cyanobacterial EPS membrane	PS, PE, PP, and PVC	EPS-biosensor EIS	0.1 µM^−1^ mM	Lowest detection limit	10^−11^ M	180

Zizhen Xiao *et al.*^186^ created a portable PEC sensor that is self-powered and incorporates both a photocathode (ITO/CuBi_2_O_4_) and photoanode (ITO/Bi_2_O_2_S). The sensor effectively utilizes the inherent variation in Fermi levels between CuBi_2_O_4_ and Bi_2_O_2_S to enhance its signals and facilitate electron flow, resulting in an impressive performance in the quantitative identification of PS MPs. This sensor is capable of detecting polystyrene microplastics (PS MPs) both sensitively and conveniently. It has a linear range of 0.5 to 1000 µg mL^−1^ and a detection limit of 0.09 µg mL^−1^ under the optimal conditions. The method exhibits strong anti-interference capacity with respect to organics and heavy metal ions. Also its accuracy can be maintained at a level exceeding 97% in the presence of interfering substances. In addition, this sensor exhibited an exceptional performance in intricate aquatic environments, thereby establishing an innovative design strategy for the development of PEC sensors for the detection of PS MPs.

A method for the on-site selective detection and quantification of microplastics in a variety of water matrices was developed by Abbas Motalebizadeh *et al.*^187^ using fluorescence-tagged peptides in conjunction with electrochemical impedance spectroscopy (EIS). Polystyrene (PS) microplastics were chosen from the various varieties of plastics present in seawater. The specific interaction of these peptides with PS spherical particles of varying diameters (0.1 to 250 µm) was confirmed using fluorometry, scanning electron microscopy (SEM), and Raman spectroscopy. The fluorescence intensity was used to determine the effects of temperature (25–65 °C), incubation time (5 and 10 min), and particle size on the plastic-peptide bonding efficacy using principal component analysis (PCA). The EIS parameters underwent a considerable change in comparison to the baseline as the plastic concentration increased. Consequently, a limit of detection (LOD) of 50 ppb (ng mL^−1^) was established for pure and tap water and 400 ppb for saline water. This sensor demonstrated exceptional efficacy in the detection of microplastics in low-ionic strength environments.

Ashab Noumani *et al.*^188^ described an electrochemical hexamethylenetetramine (HMT) sensing method employing a sensing platform constructed using a chitosan-magnesium oxide nanosheet (CHIT-MgO/NS) nanocomposite. HMT is classified as a hazardous microplastic and is employed as an additive in plastic manufacturing. Thus, it was chosen as the target analyte. To create sensing electrodes, a simple co-precipitation technique was implemented to synthesize MgO-NS. This material was subsequently combined with a 1% CHIT solution to create the CHIT-MgO-NS composite. The CHIT-MgO-NS/ITO sensing electrode was fabricated by drop-casting the nanocomposite solution onto an indium tin oxide (ITO) substrate. This electrode was used to detect HMT electrochemically using the cyclic voltammetry (CV) and differential pulse voltammetry (DPV) techniques. DPV was implemented to ascertain the limit of detection (LOD) and sensitivity. The calibration curve for HMT demonstrated a sensitivity of 12.908 µA (µM)^−1^ cm^−2^ with a detection limit of 0.03 µM and a limit of quantification (LOQ) of 0.10 µM in the range of 0.5 µM to 4.0 µM.

## 6 Summary and outlook

In summary, the growing prevalence of microplastics in both terrestrial and aquatic environments highlights the urgent necessity for effective detection, monitoring, and remediation policies. However, there is still a lack of accurate tracking, toxic exposure evaluation, and treatment techniques, despite the fact that microplastic contamination has become a global issue. Consequently, this review study looked at the toxicity, distribution, analytical techniques, health risk, and remediation technologies for microplastics. The problem of microplastics is being addressed by governments, and in the years to come, we might anticipate additional efforts to stop pollution, such as a ban on the use of plastic bags, bottles, and several other plastic items in our daily routine.

While conventional spectral analyses such as FTIR and Raman spectroscopy offer precise analysis, their reliance on complex and costly sample preparation limits their practical application. Thus, because of their ease of use, affordability, and possibility for continuous assessment, electrochemical technologies have become attractive substitutes. These techniques enhance our understanding of microplastic behaviour and support the development of innovative sensors and sustainable remediation approaches. Addressing the pollution along with the end-of-life management of microplastics requires continued research and advancement in analytical and electrochemical methodologies, which are key to mitigating their environmental footprint.

Depending on how widely they spread after exposure, microplastics may cause systemic or local immune responses. However, in other circumstances, such as genetic predisposition, environmental exposure alone can weaken the immune system and cause autoimmune diseases. Particulate matter inhalation can trigger immune cell activation, autoantibody production, and self-antigen exposure through particle translocation, cytotoxicity, inflammation, cancer, oxidative stress, and immune modulator release, all of which can result in autoimmune disorders.

Adopting eco-friendly substitutes such as organic fibre products and recyclable packaging, enforcing stringent laws on plastic manufacturing and waste disposal, and informing the public about the negative impacts of microplastics are some suggestions to lessen microplastic contamination. Therefore, there is an urgent need for national initiatives to tackle microplastic contamination as well as more experts conducting on-going research. There is currently a lack of research on microplastic pollution, evaluation, surveillance, and removal technologies.

## 7 Conclusion

Standardization and protocol development are key priorities for advancing electrochemical microplastic detection, as the lack of unified methods limits performance comparison and routine application. Future research should focus on establishing common testing procedures, calibration standards, and evaluation metrics. Improving multitarget detection is also essential, with emphasis on sensors capable of simultaneously identifying multiple microplastic types, supported by refined machine learning techniques. Moreover, integrating advanced technologies such as artificial intelligence and hybrid sensor systems can enhance data analysis, real-time monitoring, and polymer identification. Combining multiple sensing approaches may enable comprehensive microplastic characterization. Addressing these challenges is crucial for transforming laboratory-based sensors into reliable, field-deployable monitoring tools.

In the future, the successful transition of electrochemical sensors from laboratory prototypes to field-utilizable systems necessitates collaborative interdisciplinary endeavors that integrate data analytics, environmental engineering, and materials science. The ultimate objective is the development of continuous microplastic monitoring methods. By continuous innovation in electrochemical sensing technologies, it is feasible to establish networks that can monitor pollution sources, movement pathways, and environmental fate. These systems will furnish the essential data required to inform policy decisions, evaluate remediation strategies, and preserve the ecosystem and human health from the pervasive menace of microplastic contamination.

## Author contribution

Conceptualization, K. K. and A. K. S.; methodology, validation, formal analysis, data curation, software, writing – original draft preparation, K. K.; and resources, supervision, project administration, funding acquisition, writing – review & editing, validation, A. K. S.; all authors have read and agreed to the published version of the manuscript.

## Conflicts of interest

The authors declare that they have no known competing financial interests or personal relationships that could have appeared to influence the work reported in this paper.

## Data Availability

This review article utilizes previously published data, all of which have been properly cited and used with the necessary permissions. Complete details and sources of the data are provided within the manuscript.

## References

[cit1] Narayanan M. (2025). Invisible invaders: Ecotoxicological impacts of nano- and microplastics in aquatic ecosystems. Adv. Sustain. Syst..

[cit2] Jaikumar I. M., Tomson M., Meyyazhagan A., Balamuralikrishnan B., Baskaran R., Pappuswamy M., Kamyab H., Khalili E., Farajnezhad M. (2025). A comprehensive review of microplastic pollution in freshwater and marine environments. Green Anal. Chem..

[cit3] Ragu Prasath A., Sudhakar C., Selvam K. (2025). Microplastics in the environment: Types, sources, and impact on human and aquatic systems. Bioresour. Technol. Rep..

[cit4] Thanigaivel S., Kamalesh R., Ragini Y. P., Saravanan A., Vickram A. S., Abirami M., Thiruvengadam S. (2025). Microplastic pollution in marine environments: An in-depth analysis of advanced monitoring techniques, removal technologies, and future challenges. Mar. Environ. Res..

[cit5] Murugan P., Sivaperumal P., Balu S., Arya S., Atchudan R., Sundramoorthy A. K. (2023). Recent advances on the methods developed for the identification and detection of emerging contaminant microplastics: a review. RSC Adv..

[cit6] Murugan P., Jeevanandham G., Sundramoorthy A. K. (2022). Identification, interaction and detection of microplastics on fish scales (Lutjanus gibbus). Curr. Anal. Chem..

[cit7] Eriksen M., Lebreton L. C. M., Carson H. S., Thiel M., Moore C. J., Borerro J. C., Galgani F., Ryan P. G., Reisser J. (2014). Plastic pollution in the world's oceans: More than 5 trillion plastic pieces weighing over 250,000 tons afloat at sea. PLoS One.

[cit8] Kutralam-Muniasamy G., Shruti V. C., Pérez-Guevara F., Roy P. D. (2023). Microplastic diagnostics in humans: “The 3Ps” Progress, problems, and prospects. Sci. Total Environ..

[cit9] Leslie H. A., van Velzen M. J. M., Brandsma S. H., Vethaak A. D., Garcia-Vallejo J. J., Lamoree M. H. (2022). Discovery and quantification of plastic particle pollution in human blood. Environ. Int..

[cit10] Ajith N., Arumugam S., Parthasarathy S., Manupoori S., Janakiraman S. (2020). Global distribution of microplastics and its impact on marine environment-a review. Environ. Sci. Pollut. Res. Int..

[cit11] Du J., Zhou Q., Li H., Xu S., Wang C., Fu L., Tang J. (2021). Environmental distribution, transport and ecotoxicity of microplastics: A review. J. Appl. Toxicol..

[cit12] Bergmann M., Wirzberger V., Krumpen T., Lorenz C., Primpke S., Tekman M. B., Gerdts G. (2017). High quantities of microplastic in arctic deep-sea sediments from the HAUSGARTEN observatory. Environ. Sci. Technol..

[cit13] Liebezeit G., Liebezeit E. (2014). Synthetic particles as contaminants in German beers. Food Addit. Contam..

[cit14] Iñiguez M. E., Conesa J. A., Fullana A. (2017). Microplastics in Spanish table salt. Sci. Rep..

[cit15] Liebezeit G., Liebezeit E. (2013). Non-pollen particulates in honey and sugar. Food Addit. Contam..

[cit16] Liebezeit G., Liebezeit E. (2015). Origin of synthetic particles in honeys. Pol. J. Food Nutr. Sci..

[cit17] Kosuth M., Mason S. A., Wattenberg E. V. (2018). Anthropogenic contamination of tap water, beer, and sea salt. PLoS One.

[cit18] Schymanski D., Goldbeck C., Humpf H.-U., Fürst P. (2018). Analysis of microplastics in water by micro-Raman spectroscopy: Release of plastic particles from different packaging into mineral water. Water Res..

[cit19] Hernandez L. M., Xu E. G., Larsson H. C. E., Tahara R., Maisuria V. B., Tufenkji N. (2019). Plastic teabags release billions of microparticles and nanoparticles into tea. Environ. Sci. Technol..

[cit20] Van Cauwenberghe L., Janssen C. R. (2014). Microplastics in bivalves cultured for human consumption. Environ. Pollut..

[cit21] Lebreton L. C. M., van der Zwet J., Damsteeg J.-W., Slat B., Andrady A., Reisser J. (2017). River plastic emissions to the world's oceans. Nat. Commun..

[cit22] Thompson R. C., Olsen Y., Mitchell R. P., Davis A., Rowland S. J., John A. W. G., McGonigle D., Russell A. E. (2004). Lost at sea: where is all the plastic?. Science.

[cit23] Scheurer M., Bigalke M. (2018). Microplastics in Swiss floodplain soils. Environ. Sci. Technol..

[cit24] Jolaosho T. L. (2025). *et al.*, Microplastics in freshwater and marine ecosystems: Occurrence, characterization, sources, distribution dynamics, fate, transport processes, potential mitigation strategies, and policy interventions. Ecotoxicol. Environ. Saf..

[cit25] Berkel C., Ozbek O. (2024). Methods used in the identification plastics from water environments. South Afr. J. Chem. Eng..

[cit26] Guo X., Wang J. (2019). The chemical behaviors of microplastics in marine environment: A review. Mar. Pollut. Bull..

[cit27] Anagnosti L., Varvaresou A., Pavlou P., Protopapa E., Carayanni V. (2021). Worldwide actions against plastic pollution from microbeads and microplastics in cosmetics focusing on European policies. Has the issue been handled effectively?. Mar. Pollut. Bull..

[cit28] Liu J., Liang Z. (2025). Microplastic migration and transformation pathways and exposure health risks. Environ. Pollut..

[cit29] Anderson P. J., Warrack S., Langen V., Challis J. K., Hanson M. L., Rennie M. D. (2017). Microplastic contamination in Lake Winnipeg, Canada. Environ. Pollut..

[cit30] He S., Jia M., Xiang Y., Song B., Xiong W., Cao J., Peng H., Yang Y., Wang W., Yang Z., Zeng G. (2022). Biofilm on microplastics in aqueous environment: Physicochemical properties and environmental implications. J. Hazard. Mater..

[cit31] Alomar C., Estarellas F., Deudero S. (2016). Microplastics in the Mediterranean Sea: Deposition in coastal shallow sediments, spatial variation and preferential grain size. Mar. Environ. Res..

[cit32] Fendall L. S., Sewell M. A. (2009). Contributing to marine pollution by washing your face: microplastics in facial cleansers. Mar. Pollut. Bull..

[cit33] Fadare O. O., Wan B., Guo L.-H., Zhao L. (2020). Microplastics from consumer plastic food containers: Are we consuming it?. Chemosphere.

[cit34] Fadare O. O., Okoffo E. D. (2020). Covid-19 face masks: A potential source of microplastic fibers in the environment. Sci. Total Environ..

[cit35] Yang Z., Lü F., Zhang H., Wang W., Shao L., Ye J., He P. (2021). Is incineration the terminator of plastics and microplastics?. J. Hazard. Mater..

[cit36] Čulin J., Bielić T. (2016). Plastic pollution from ships, Pomor. Zb.

[cit37] Rolsky C., Kelkar V., Driver E., Halden R. U. (2020). Municipal sewage sludge as a source of microplastics in the environment. Curr. Opin. Environ. Sci. Health.

[cit38] Hale R. C., Seeley M. E., La Guardia M. J., Mai L., Zeng E. Y. (2020). A global perspective on microplastics. J. Geophys. Res. Oceans.

[cit39] Rochman C. M. (2018). Microplastics research-from sink to source. Science.

[cit40] Guerranti C., Martellini T., Perra G., Scopetani C., Cincinelli A. (2019). Microplastics in cosmetics: Environmental issues and needs for global bans. Environ. Toxicol. Pharmacol..

[cit41] Carney Almroth B. M., Åström L., Roslund S., Petersson H., Johansson M., Persson N.-K. (2018). Quantifying shedding of synthetic fibers from textiles; a source of microplastics released into the environment. Environ. Sci. Pollut. Res. Int..

[cit42] Li J., Zhang K., Zhang H. (2018). Adsorption of antibiotics on microplastics. Environ. Pollut..

[cit43] Karbalaei S., Golieskardi A., Hamzah H. B., Abdulwahid S., Hanachi P., Walker T. R., Karami A. (2019). Abundance and characteristics of microplastics in commercial marine fish from Malaysia. Mar. Pollut. Bull..

[cit44] Naji A., Esmaili Z., Khan F. R. (2017). Plastic debris and microplastics along the beaches of the Strait of Hormuz, Persian Gulf. Mar. Pollut. Bull..

[cit45] Good T. P., June J. A., Etnier M. A., Broadhurst G. (2010). Derelict fishing nets in Puget Sound and the Northwest Straits: patterns and threats to marine fauna. Mar. Pollut. Bull..

[cit46] Peng G., Xu P., Zhu B., Bai M., Li D. (2018). Microplastics in freshwater river sediments in Shanghai, China: A case study of risk assessment in mega-cities. Environ. Pollut..

[cit47] Calero M., Godoy V., Quesada L., Martín-Lara M. Á. (2021). Green strategies for microplastics reduction. Curr. Opin. Green Sustain. Chem..

[cit48] Wong J. K. H., Lee K. K., Tang K. H. D., Yap P.-S. (2020). Microplastics in the freshwater and terrestrial environments: Prevalence, fates, impacts and sustainable solutions. Sci. Total Environ..

[cit49] Edo C., González-Pleiter M., Leganés F., Fernández-Piñas F., Rosal R. (2020). Fate of microplastics in wastewater treatment plants and their environmental dispersion with effluent and sludge. Environ. Pollut..

[cit50] Auta H. S., Emenike C. U., Fauziah S. H. (2017). Distribution and importance of microplastics in the marine environment: A review of the sources, fate, effects, and potential solutions. Environ. Int..

[cit51] Facciolà A., Visalli G., Pruiti Ciarello M., Di Pietro A. (2021). Newly emerging airborne pollutants: Current knowledge of health impact of micro and nanoplastics. Int. J. Environ. Res. Public Health.

[cit52] Kwon J.-H., Kim J.-W., Pham T. D., Tarafdar A., Hong S., Chun S.-H., Lee S.-H., Kang D.-Y., Kim J.-Y., Kim S.-B., Jung J. (2020). Microplastics in food: A review on analytical methods and challenges. Int. J. Environ. Res. Public Health.

[cit53] Bouwmeester H., Hollman P. C. H., Peters R. J. B. (2015). Potential health impact of environmentally released micro- and nanoplastics in the human food production chain: Experiences from nanotoxicology. Environ. Sci. Technol..

[cit54] Xu M., Halimu G., Zhang Q., Song Y., Fu X., Li Y., Li Y., Zhang H. (2019). Internalization and toxicity: A preliminary study of effects of nanoplastic particles on human lung epithelial cell. Sci. Total Environ..

[cit55] Fournier S. B., D'Errico J. N., Adler D. S., Kollontzi S., Goedken M. J., Fabris L., Yurkow E. J., Stapleton P. A. (2020). Nanopolystyrene translocation and fetal deposition after acute lung exposure during late-stage pregnancy. Part. Fibre Toxicol..

[cit56] Ragusa A., Svelato A., Santacroce C., Catalano P., Notarstefano V., Carnevali O., Papa F., Rongioletti M. C. A., Baiocco F., Draghi S., D'Amore E., Rinaldo D., Matta M., Giorgini E. (2021). Plasticenta: First evidence of microplastics in human placenta. Environ. Int..

[cit57] Cunningham E. M., Ehlers S. M., Dick J. T. A., Sigwart J. D., Linse K., Dick J. J., Kiriakoulakis K. (2020). High abundances of microplastic pollution in deep-sea sediments: Evidence from Antarctica and the Southern Ocean. Environ. Sci. Technol..

[cit58] Napper I. E., Davies B. F. R., Clifford H., Elvin S., Koldewey H. J., Mayewski P. A., Miner K. R., Potocki M., Elmore A. C., Gajurel A. P., Thompson R. C. (2020). Reaching new heights in plastic pollution—preliminary findings of microplastics on mount Everest. One Earth.

[cit59] Sana S. S., Dogiparthi L. K., Gangadhar L., Chakravorty A., Abhishek N. (2020). Effects of microplastics and nanoplastics on marine environment and human health. Environ. Sci. Pollut. Res. Int..

[cit60] Campanale C. (2020). *et al.*, A detailed review study on potential effects of microplastics and additives of concern on human health. Int. J. Environ. Res. Publ. Health.

[cit61] de Souza Machado A. A., Lau C. W., Till J., Kloas W., Lehmann A., Becker R., Rillig M. C. (2018). Impacts of microplastics on the soil biophysical environment. Environ. Sci. Technol..

[cit62] de Souza Machado A. A., Kloas W., Zarfl C., Hempel S., Rillig M. C. (2018). Microplastics as an emerging threat to terrestrial ecosystems. Glob. Chang. Biol..

[cit63] Huerta Lwanga E., Mendoza Vega J., Ku Quej V., Chi J. de L. A., Sanchez Del Cid L., Chi C., Escalona Segura G., Gertsen H., Salánki T., van der Ploeg M., Koelmans A. A., Geissen V. (2017). Field evidence for transfer of plastic debris along a terrestrial food chain. Sci. Rep..

[cit64] Ng E.-L., Huerta Lwanga E., Eldridge S. M., Johnston P., Hu H.-W., Geissen V., Chen D. (2018). An overview of microplastic and nanoplastic pollution in agroecosystems. Sci. Total Environ..

[cit65] Zhao S., Zhu L., Li D. (2016). Microscopic anthropogenic litter in terrestrial birds from Shanghai, China: Not only plastics but also natural fibers. Sci. Total Environ..

[cit66] Guo J.-J., Huang X.-P., Xiang L., Wang Y.-Z., Li Y.-W., Li H., Cai Q.-Y., Mo C.-H., Wong M.-H. (2020). Source, migration and toxicology of microplastics in soil. Environ. Int..

[cit67] de Souza Machado A. A., Lau C. W., Kloas W., Bergmann J., Bachelier J. B., Faltin E., Becker R., Görlich A. S., Rillig M. C. (2019). Microplastics can change soil properties and affect plant performance. Environ. Sci. Technol..

[cit68] Huerta Lwanga E., Gertsen H., Gooren H., Peters P., Salánki T., van der Ploeg M., Besseling E., Koelmans A. A., Geissen V. (2016). Microplastics in the Terrestrial Ecosystem: Implications for Lumbricus terrestris (Oligochaeta, Lumbricidae). Environ. Sci. Technol..

[cit69] Lin D., Yang G., Dou P., Qian S., Zhao L., Yang Y., Fanin N. (1934). Microplastics negatively affect soil fauna but stimulate microbial activity: insights from a field-based microplastic addition experiment. Proc. Biol. Sci..

[cit70] Cesa F. S., Turra A., Checon H. H., Leonardi B., Baruque-Ramos J. (2020). Laundering and textile parameters influence fibers release in household washings. Environ. Pollut..

[cit71] Schirinzi G. F., Pérez-Pomeda I., Sanchís J., Rossini C., Farré M., Barceló D. (2017). Cytotoxic effects of commonly used nanomaterials and microplastics on cerebral and epithelial human cells. Environ. Res..

[cit72] Ahmad M., Li J.-L., Wang P.-D., Hozzein W. N., Li W.-J. (2020). Environmental perspectives of microplastic pollution in the aquatic environment: a review, Mar. Life Sci. Technol..

[cit73] Amobonye A., Bhagwat P., Singh S., Pillai S. (2021). Plastic biodegradation: Frontline microbes and their enzymes. Sci. Total Environ..

[cit74] Jones N. R. (2024). *et al.*, Identifying laboratory sources of microplastic and nanoplastic contamination from the air, water, and consumables. J. Hazard Mater..

[cit75] Yadav P. (2025). *et al.*, Microbial degradation of microplastics: Effectiveness, challenges, and sustainable solutions. Curr. Res. Microb. Sci..

[cit76] Ge J. (2022). *et al.*, Photocatalytic degradation of (micro) plastics using TiO2-based and other catalysts: Properties, influencing factor, and mechanism. Environ. Res..

[cit77] Andrady A. L. (2022). *et al.*, Oxidation and fragmentation of plastics in a changing environment; from UV-radiation to biological degradation. Sci. Total Environ..

[cit78] Dey S. (2024). *et al.*, Degradation of plastics waste and its effects on biological ecosystems: A scientific analysis and comprehensive review. Biomed.
Mater. & Devices.

[cit79] Im D., Gavande V., Lee H. Y., Lee W.-K. (2023). Influence of Molecular Weight on the Enzymatic Degradation of PLA Isomer Blends by a Langmuir System. Materials.

[cit80] Lucas N., Bienaime C., Belloy C., Queneudec M., Silvestre F., Nava-Saucedo J.-E. (2008). Polymer biodegradation: mechanisms and estimation techniques. Chemosphere.

[cit81] Shah A. A., Hasan F., Hameed A., Ahmed S. (2008). Biological degradation of plastics: a comprehensive review. Biotechnol. Adv..

[cit82] Sivan A. (2011). New perspectives in plastic biodegradation. Curr. Opin. Biotechnol..

[cit83] Balasubramanian V., Natarajan K., Hemambika B., Ramesh N., Sumathi C. S., Kottaimuthu R., Rajesh Kannan V. (2010). High-density polyethylene (HDPE)-degrading potential bacteria from marine ecosystem of Gulf of Mannar, India. Lett. Appl. Microbiol..

[cit84] Tribedi P., Sil A. K. (2014). Cell surface hydrophobicity: a key component in the degradation of polyethylene succinate by Pseudomonas sp. AKS2, J. Appl. Microbiol..

[cit85] Nauendorf A., Krause S., Bigalke N. K., Gorb E. V., Gorb S. N., Haeckel M., Wahl M., Treude T. (2016). Microbial colonization and degradation of polyethylene and biodegradable plastic bags in temperate fine-grained organic-rich marine sediments. Mar. Pollut. Bull..

[cit86] Wilkes R. A., Aristilde L. (2017). Degradation and metabolism of synthetic plastics and associated products by Pseudomonas sp.: capabilities and challenges. J. Appl. Microbiol..

[cit87] Kjeldsen A. (2018). *et al.*, A review of standards for biodegradable plastics. Ind. Biotechnol. Innov. Cent.

[cit88] Laycock B., Nikolić M., Colwell J. M., Gauthier E., Halley P., Bottle S., George G. (2017). Lifetime prediction of biodegradable polymers. Prog. Polym. Sci..

[cit89] Ali S. S., Elsamahy T., Koutra E., Kornaros M., El-Sheekh M., Abdelkarim E. A., Zhu D., Sun J. (2021). Degradation of conventional plastic wastes in the environment: A review on current status of knowledge and future perspectives of disposal. Sci. Total Environ..

[cit90] Filiciotto L., Rothenberg G. (2021). Biodegradable plastics: Standards, policies, and impacts. ChemSusChem.

[cit91] Matjašič T., Simčič T., Medvešček N., Bajt O., Dreo T., Mori N. (2021). Critical evaluation of biodegradation studies on synthetic plastics through a systematic literature review. Sci. Total Environ..

[cit92] Hou L., Xi J., Liu J., Wang P., Xu T., Liu T., Qu W., Lin Y. B. (2022). Biodegradability of polyethylene mulching film by two Pseudomonas bacteria and their potential degradation mechanism. Chemosphere.

[cit93] Park S. Y., Kim C. G. (2019). Biodegradation of micro-polyethylene particles by bacterial colonization of a mixed microbial consortium isolated from a landfill site. Chemosphere.

[cit94] Kim H. R., Lee H. M., Yu H. C., Jeon E., Lee S., Li J., Kim D.-H. (2020). Biodegradation of Polystyrene by Pseudomonas sp. Isolated from the Gut of Superworms (Larvae of Zophobas atratus). Environ. Sci. Technol..

[cit95] Auta H. S., Emenike C. U., Jayanthi B., Fauziah S. H. (2018). Growth kinetics and biodeterioration of polypropylene microplastics by Bacillus sp. and Rhodococcus sp. isolated from mangrove sediment. Mar. Pollut. Bull..

[cit96] Sánchez C. (2020). Fungal potential for the degradation of petroleum-based polymers: An overview of macro- and microplastics biodegradation. Biotechnol. Adv..

[cit97] Gao R., Liu R., Sun C. (2022). A marine fungus Alternaria alternata FB1 efficiently degrades polyethylene. J. Hazard. Mater..

[cit98] Yan N., Fan C., Chen Y., Hu Z. (2016). The potential for microalgae as bioreactors to produce pharmaceuticals. Int. J. Mol. Sci..

[cit99] Vimal Kumar R., Kanna G. R., Elumalai S. (2017). Biodegradation of polyethylene by green photosynthetic microalgae. J. Bioremediat. Biodegrad..

[cit100] Chia W. Y., Ying Tang D. Y., Khoo K. S., Kay Lup A. N., Chew K. W. (2020). Nature's fight against plastic pollution: Algae for plastic biodegradation and bioplastics production. Environ. Sci. Ecotechnol..

[cit101] Akram M. A., Savitha R., Kinsella G. K., Nolan K., Ryan B. J., Henehan G. T. (2024). Microbial and Enzymatic Biodegradation of Plastic Waste for a Circular Economy. Appl. Sci..

[cit102] Du H., Xie Y., Wang J. (2021). Microplastic degradation methods and corresponding degradation mechanism: Research status and future perspectives. J. Hazard. Mater..

[cit103] Kang J., Zhou L., Duan X., Sun H., Ao Z., Wang S. (2019). Degradation of cosmetic microplastics via functionalized carbon nanosprings. Matter.

[cit104] Tofa T. S., Kunjali K. L., Paul S., Dutta J. (2019). Visible light photocatalytic degradation of microplastic residues with zinc oxide nanorods. Environ. Chem. Lett..

[cit105] Gewert B., Plassmann M. M., MacLeod M. (2015). Pathways for degradation of plastic polymers floating in the marine environment. Environ. Sci. Process. Impacts.

[cit106] Song Y. K., Hong S. H., Jang M., Han G. M., Jung S. W., Shim W. J. (2017). Combined effects of UV exposure duration and mechanical abrasion on microplastic fragmentation by polymer type. Environ. Sci. Technol..

[cit107] Al-Madhagi H. (2025). Microplastics toxicology and bioremediation strategies for a sustainable future: a comprehensive review. Green Chem. Lett. Rev..

[cit108] Zhu H. (2025). *et al.*, Advances in Plasma-Assisted Degradation of Plastic Pollutants: From Reaction Mechanisms to Engineering Applications. J. Environ. Chem. Eng..

[cit109] Nhan N. T., Luu T. L. (2025). Current status of using adsorbent nanomaterials for removing microplastics from water supply systems: a mini review. Beilstein J. Nanotechnol..

[cit110] Honarmandrad Z., Kaykhaii M., Gębicki J. (2023). Microplastics removal from aqueous environment by metal organic frameworks. BMC Chem..

[cit111] Barari F. (2025). *et al.*, Advances in metal-organic frameworks for microplastic removal from aquatic environments: mechanisms and performance insights. Results Chem..

[cit112] Dey T. K. (2023). *et al.*, Metal-organic framework
membrane for waterborne micro/nanoplastics treatment. Chem. Eng. J..

[cit113] Hidalgo-Ruz V., Gutow L., Thompson R. C., Thiel M. (2012). Microplastics in the marine environment: a review of the methods used for identification and quantification. Environ. Sci. Technol..

[cit114] Filella M. (2015). Questions of size and numbers in environmental research on microplastics: methodological and conceptual aspects. Environ. Chem..

[cit115] Hanvey J. S., Lewis P. J., Lavers J. L., Crosbie N. D., Pozo K., Clarke B. O. (2017). A review of analytical techniques for quantifying microplastics in sediments. Anal. Methods.

[cit116] Mossotti R., Dalla Fontana G., Anceschi A., Gasparin E., Battistini T. (2021). Preparation and analysis of standards containing microfilaments/microplastic with fibre shape. Chemosphere.

[cit117] Abbasi S. (2021). Prevalence and physicochemical characteristics of microplastics in the sediment and water of Hashilan Wetland, a national heritage in NW Iran. Environ. Technol. Innov..

[cit118] Sorasan C., Edo C., González-Pleiter M., Fernández-Piñas F., Leganés F., Rodríguez A., Rosal R. (2021). Generation of nanoplastics during the photoageing of low-density polyethylene. Environ. Pollut..

[cit119] Bittelli M., Pellegrini S., Olmi R., Andrenelli M. C., Simonetti G., Borrelli E., Morari F. (2022). Experimental evidence of laser diffraction accuracy for particle size analysis. Geoderma.

[cit120] Blott S. J., Croft D. J., Pye K., Saye S. E., Wilson H. E. (2004). Particle size analysis by laser diffraction. Geol. Soc. Spec. Publ..

[cit121] Kedzierski M., Falcou-Préfol M., Kerros M. E., Henry M., Pedrotti M. L., Bruzaud S. (2019). A machine learning algorithm for high throughput identification of FTIR spectra: Application on microplastics collected in the Mediterranean Sea. Chemosphere.

[cit122] Morgado V., Gomes L., Bettencourt da Silva R. J. N., Palma C. (2021). Validated spreadsheet for the identification of PE, PET, PP and PS microplastics by micro-ATR-FTIR spectra with known uncertainty. Talanta.

[cit123] Vinay Kumar B. N., Löschel L. A., Imhof H. K., Löder M. G. J., Laforsch C. (2021). Analysis of microplastics of a broad size range in commercially important mussels by combining FTIR and Raman spectroscopy approaches. Environ. Pollut..

[cit124] Li J., Liu H., Paul Chen J. (2018). Microplastics in freshwater systems: A review on occurrence, environmental effects, and methods for microplastics detection. Water Res..

[cit125] Nava V., Frezzotti M. L., Leoni B. (2021). Raman spectroscopy for the analysis of microplastics in aquatic systems. Appl. Spectrosc..

[cit126] Stiles P. L., Dieringer J. A., Shah N. C., Van Duyne R. P. (2008). Surface-enhanced Raman spectroscopy. Annu. Rev. Anal. Chem..

[cit127] Wang Y., Yan B., Chen L. (2013). SERS tags: novel optical nanoprobes for bioanalysis. Chem. Rev..

[cit128] Pang S., Yang T., He L. (2016). Review of surface enhanced Raman spectroscopic (SERS) detection of synthetic chemical pesticides. Trends Analyt. Chem..

[cit129] Janči T., Valinger D., Gajdoš Kljusurić J., Mikac L., Vidaček S., Ivanda M. (2017). Determination of histamine in fish by Surface Enhanced Raman Spectroscopy using silver colloid SERS substrates. Food Chem..

[cit130] Hakonen A., Andersson P. O., Stenbæk Schmidt M., Rindzevicius T., Käll M. (2015). Explosive and chemical threat detection by surface-enhanced Raman scattering: a review. Anal. Chim. Acta.

[cit131] Xu G., Cheng H., Jones R., Feng Y., Gong K., Li K., Fang X., Tahir M. A., Valev V. K., Zhang L. (2020). Surface-enhanced Raman spectroscopy facilitates the detection of microplastics <1 µm in the environment. Environ. Sci. Technol..

[cit132] Jeon Y., Kim D., Kwon G., Lee K., Oh C.-S., Kim U.-J., You J. (2021). Detection of nanoplastics based on surface-enhanced Raman scattering with silver nanowire arrays on regenerated cellulose films. Carbohydr. Polym..

[cit133] Caldwell J., Taladriz-Blanco P., Rothen-Rutishauser B., Petri-Fink A. (2021). Detection of sub-micro- and nanoplastic particles on gold nanoparticle-based substrates through surface-enhanced Raman scattering (SERS) spectroscopy. Nanomaterials.

[cit134] Caldwell J., Lehner R., Balog S., Rhême C., Gao X., Septiadi D., Weder C., Petri-Fink A., Rothen-Rutishauser B. (2021). Fluorescent plastic nanoparticles to track their interaction and fate in physiological environments. Environ. Sci. Nano.

[cit135] Nie X.-L., Liu H.-L., Pan Z.-Q., Ahmed S. A., Shen Q., Yang J.-M., Pan J.-B., Pang J., Li C.-Y., Xia X.-H., Wang K. (2019). Recognition of plastic nanoparticles using a single gold nanopore fabricated at the tip of a glass nanopipette. Chem. Commun..

[cit136] Lv L., He L., Jiang S., Chen J., Zhou C., Qu J., Lu Y., Hong P., Sun S., Li C. (2020). In situ surface-enhanced Raman spectroscopy for detecting microplastics and nanoplastics in aquatic environments. Sci. Total Environ..

[cit137] Zhou X.-X., Liu R., Hao L.-T., Liu J.-F. (2021). Identification of polystyrene nanoplastics using surface enhanced Raman spectroscopy. Talanta.

[cit138] Hu R., Zhang K., Wang W., Wei L., Lai Y. (2022). Quantitative and sensitive analysis of polystyrene nanoplastics down to 50 nm by surface-enhanced Raman spectroscopy in water. J. Hazard. Mater..

[cit139] Lee C.-H., Fang J. K.-H. (2022). The onset of surface-enhanced Raman scattering for single-particle detection of submicroplastics. J. Environ. Sci..

[cit140] Laborda F., Gimenez-Ingalaturre A. C., Bolea E., Castillo J. R. (2019). Single particle inductively coupled plasma mass spectrometry as screening tool for detection of particles. Spectrochim. Acta, Part B.

[cit141] Huang Y., Zhao Y., Wang J., Zhang M., Jia W., Qin X. (2019). LDPE microplastic films alter microbial community composition and enzymatic activities in soil. Environ. Pollut..

[cit142] Keller A. A., Huang Y., Nelson J. (2018). Detection of Nanoparticles in Edible Plant Tissues Exposed to Nano-Copper Using Single-Particle ICP MS. J. Nanopart. Res..

[cit143] Cervantes Avilés P., Huang Y., Keller A. A. (2019). Incidence and Persistence of Silver Nanoparticles throughout the Wastewater Treatment Process. Water Res..

[cit144] Lamana J. (2020). A Novel Strategy for the Detection and Quantification of Nanoplastics by Single Particle Inductively Coupled Plasma Mass Spectrometry (ICP MS). Anal. Chem..

[cit145] Lai Y. (2021). Counting Nanoplastics in Environmental Waters by Single Particle Inductively Coupled Plasma Mass Spectroscopy after Cloud Point Extraction and In Situ Labeling of Gold Nanoparticles. Environ. Sci. Technol..

[cit146] Bolea Fernandez E. (2020). Detection of Microplastics Using Inductively Coupled Plasma-Mass Spectrometry (ICP MS) Operated in Single Event Mode. J. Anal. At. Spectrom..

[cit147] Laborda F., Trujillo C., Lobinski R. (2021). Analysis of Microplastics in Consumer Products by Single Particle Inductively Coupled Plasma Mass Spectrometry Using the Carbon 13 Isotope. Talanta.

[cit148] Nuelle M.-T., Dekiff J. H., Remy D., Fries E. (2014). A new analytical approach for monitoring microplastics in marine sediments. Environ. Pollut..

[cit149] Fischer M., Scholz-Böttcher B. M. (2017). Simultaneous trace identification and quantification of common types of microplastics in environmental samples by pyrolysis-gas chromatography-mass spectrometry. Environ. Sci. Technol..

[cit150] Käppler A., Fischer M., Scholz-Böttcher B. M., Oberbeckmann S., Labrenz M., Fischer D., Eichhorn K.-J., Voit B. (2018). Comparison of µ-ATR-FTIR spectroscopy and py-GCMS as identification tools for microplastic particles and fibers isolated from river sediments. Anal. Bioanal. Chem..

[cit151] Dümichen E., Eisentraut P., Bannick C. G., Barthel A.-K., Senz R., Braun U. (2017). Fast identification of microplastics in complex environmental samples by a thermal degradation method. Chemosphere.

[cit152] Zhang J., Wang L., Kannan K. (2021). Quantitative analysis of polyethylene terephthalate and polycarbonate microplastics in sediment collected from South Korea, Japan and the USA. Chemosphere.

[cit153] Zhang J., Wang L., Halden R. U., Kannan K. (2019). Polyethylene terephthalate and polycarbonate microplastics in sewage sludge collected from the United States. Environ. Sci. Technol. Lett..

[cit154] Peng C., Tang X., Gong X., Dai Y., Sun H., Wang L. (2020). Development and application of a mass spectrometry method for quantifying nylon microplastics in environment. Anal. Chem..

[cit155] Wang L., Zhang J., Hou S., Sun H. (2017). A simple method for quantifying polycarbonate and polyethylene terephthalate microplastics in environmental samples by liquid chromatography–tandem mass spectrometry. Environ. Sci. Technol. Lett..

[cit156] Majewsky M., Bitter H., Eiche E., Horn H. (2016). Determination of microplastic polyethylene (PE) and polypropylene (PP) in environmental samples using thermal analysis (TGA-DSC). Sci. Total Environ..

[cit157] David J., Steinmetz Z., Kučerík J., Schaumann G. E. (2018). Quantitative analysis of poly(ethylene terephthalate) microplastics in soil via thermogravimetry-mass spectrometry. Anal. Chem..

[cit158] Huppertsberg S., Knepper T. P. (2018). Instrumental analysis of microplastics-benefits and challenges. Anal. Bioanal. Chem..

[cit159] Castañeda R. A., Avlijas S., Simard M. A., Ricciardi A. (2014). Microplastic pollution in St. Lawrence River sediments. Can. J. Fish. Aquat. Sci..

[cit160] Zainuddin Z. (2020). Syuhada, Study of analysis method on microplastic identification in bottled drinking water. Macromol. Symp..

[cit161] Rodríguez Chialanza M., Sierra I., Pérez Parada A., Fornaro L. (2018). Identification and quantitation of semi-crystalline microplastics using image analysis and differential scanning calorimetry. Environ. Sci. Pollut. Res. Int..

[cit162] Erni Cassola G. (2017). Lost, but Found with Nile Red: A Novel Method for Detecting and Quantifying Small Microplastics (1 mm to 20 µm) in Environmental Samples. Environ. Sci. Technol..

[cit163] Simmerman C. B., Coleman Wasik J. K. (2020). The effect of urban point source contamination on microplastic levels in water and organisms in a cold-water stream. Limnol. Oceanogr. Lett..

[cit164] Zhang J., Tian K., Lei C., Min S. (2018). Identification and quantification of microplastics in table sea salts using micro-NIR imaging methods. Anal. Methods.

[cit165] Paul A., Wander L., Becker R., Goedecke C., Braun U. (2019). High-throughput NIR spectroscopic (NIRS) detection of microplastics in soil. Environ. Sci. Pollut. Res. Int..

[cit166] Butement J. T. (2024). *et al.*, Discrimination of microplastics and phytoplankton using impedance cytometry. ACS Sensors.

[cit167] Rathore C., Saha M., Gupta P., Kumar M., Naik A., de Boer J. (2023). Standardization of micro-FTIR methods and applicability for the detection and identification of microplastics in environmental matrices. Sci. Total Environ..

[cit168] Willans M., Szczecinski E., Roocke C., Williams S., Timalsina S., Vongsvivut J., McIlwain J., Naderi G., Linge K. L., Hackett M. J. (2023). Development of a rapid detection protocol for microplastics using reflectance-FTIR spectroscopic imaging and multivariate classification. Env. Sci. Adv..

[cit169] Mikac L., Rigó I., Himics L., Tolić A., Ivanda M., Veres M. (2023). Surface-enhanced Raman spectroscopy for the detection of microplastics. Appl. Surf. Sci..

[cit170] Kniggendorf A.-K., Wetzel C., Roth B. (2019). Microplastics detection in streaming tap water with Raman spectroscopy. Sensors.

[cit171] Jin N., Song Y., Ma R., Li J., Li G., Zhang D. (2022). Characterization and identification of microplastics using Raman spectroscopy coupled with multivariate analysis. Anal. Chim. Acta.

[cit172] Vincent T. , Asogan D. and Kutscher D., Analysis of microplastics as emerging contaminants using single particle ICP-MS, https://brjac.com.br/artigos/brjac-41-thermo-report-AN001223.pdf, accessed November 3, 2025

[cit173] Gniadek M., Dąbrowska A. (2019). The marine nano- and microplastics characterisation by SEM-EDX: The potential of the method in comparison with various physical and chemical approaches. Mar. Pollut. Bull..

[cit174] Yoo H., Kim M., Lee Y., Park J., Lee H., Song Y.-C., Ro C.-U. (2023). Novel single-particle analytical technique for inhalable airborne microplastic particles by the combined use of fluorescence microscopy, Raman microspectrometry, and SEM/EDX. Anal. Chem..

[cit175] Gomiero A., Øysæd K. B., Palmas L., Skogerbø G. (2021). Application of GCMS-pyrolysis to estimate the levels of microplastics in a drinking water supply system. J. Hazard. Mater..

[cit176] Santos L. H. M. L., Insa S., Arxé M., Buttiglieri G., Rodríguez-Mozaz S., Barceló D. (2023). Analysis of microplastics in the environment: Identification and quantification of trace levels of common types of plastic polymers using pyrolysis-GC/MS. MethodsX.

[cit177] Kumar D. H. L. (2025). *et al.*, Electrochemical approaches for detecting micro and nano-plastics in different environmental matrices. Int. J. Electrochem. Sci..

[cit178] Shimizu K., Sokolov S. V., Kätelhön E., Holter J., Young N. P., Compton R. G. (2017). *In situ* Detection of Microplastics: Single Microparticle-electrode Impacts. Electroanalysis.

[cit179] Colson B. C., Michel A. P. M. (2021). Flow-through quantification of microplastics using impedance spectroscopy. ACS Sens.

[cit180] Gongi W., Touzi H., Sadly I., Ben Ouada H., Tamarin O., Ben Ouada H. (2022). A novel impedimetric sensor based on cyanobacterial extracellular polymeric substances for microplastics detection. J. Polym. Environ..

[cit181] Wang S., Xu M., Jin B., Wünsch U. J., Su Y., Zhang Y. (2022). Electrochemical and microbiological response of exoelectrogenic biofilm to polyethylene microplastics in water. Water Res..

[cit182] Du H., Chen G., Wang J. (2023). Highly selective electrochemical impedance spectroscopy-based graphene electrode for rapid detection of microplastics. Sci. Total Environ..

[cit183] Lee C., Han S., Park J. H. (2024). Electrochemical detection of microplastics in water using ultramicroelectrodes. Chemosensors.

[cit184] Surucu O. (2025). An electrochemical detection method for polyvinyl chloride microplastics in water bodies. J. Mol. Struct..

[cit185] Shabib A., Maraqa M. A., Mohammad A. F., Awwad F. (2025). Design, fabrication, and application of electrochemical sensors for microplastic detection: a state-of-the-art review and future perspectives. Environ. Sci. Eur..

[cit186] Xiao Z., Chen Y., Zhang Y. (2025). Self-powered portable photoelectrochemical sensor based on dual-photoelectrode for microplastics detection. Environ. Res..

[cit187] Motalebizadeh A., Fardindoost S., Hoorfar M. (2024). Selective on-site detection and quantification of polystyrene microplastics in water using fluorescence-tagged peptides and electrochemical impedance spectroscopy. J. Hazard. Mater..

[cit188] Noumani A., Verma D., Kaushik A., Khosla A., Solanki P. R. (2024). Electrochemically microplastic detection using chitosan-magnesium oxide nanosheet. Environ. Res..

